# An integrated annotation strategy for the phytochemical characterization of Xie-Bai-San decoction based on UPLC-Q Exactive Orbitrap HRMS, multi-database screening, and feature-based molecular networking

**DOI:** 10.3389/fchem.2026.1848728

**Published:** 2026-06-17

**Authors:** Hongjiao Chen, Kun Zhang, Yujiao Huang, Ting Chen, Yinming Zhao, Fengyu Jin, Jianjun Zhang, Linyuan Wang

**Affiliations:** 1 School of Chinese Materia Medica, Beijing University of Chinese Medicine, Beijing, China; 2 School of Traditional Chinese Medicine, Beijing University of Chinese Medicine, Beijing, China

**Keywords:** compound annotation, FBMN, spectral databases, UPLC–Q Exactive Orbitrap HRMS, Xie-Bai-San decoction

## Abstract

**Introduction:**

Characterizing plant-derived compounds in complex botanical formulations remains challenging because of matrix complexity, structurally related analogues, and frequent isomerism. Xie-Bai-San decoction (XBSD), a traditional herbal formula composed of Cortex Mori, Cortex Lycii, and Radix et Rhizoma Glycyrrhizae, represents a complex botanical system requiring comprehensive phytochemical characterization.

**Methods:**

An integrated phytochemical annotation strategy was developed using UPLC-Q Exactive Orbitrap HRMS combined with an automated data processing workflow incorporating an in-house library, public databases, and feature-based molecular networking (FBMN). Compound characterization was further supported by reference standard validation, MS/MS spectral interpretation, literature comparison, and confidence-level assignment.

**Results:**

A total of 218 compounds were annotated or tentatively characterized in XBSD, including 80 flavonoids, 35 organic acids, 23 amides, 21 terpenoids, 17 coumarins, 8 saccharides, 6 alkaloids, 5 stilbenes, 3 cyclic peptides, and 20 other constituents. Among them, 74 compounds were reported from XBSD for the first time, and 13 compounds were unambiguously identified using authentic reference standards. Representative MS/MS fragmentation features were elucidated to guide the annotation of structurally related analogues. FBMN enabled the visual clustering of molecular families, facilitating rapid structural inference and improving confidence in analogue annotation, particularly for isomers and low-abundance compounds.

**Discussion:**

This study provides a comprehensive chemical profile of XBSD and demonstrates that the integrated UPLC-HRMS-based workflow improves the efficiency, reliability, and transparency of phytochemical annotation in complex traditional herbal formulations. The proposed strategy may serve as a practical analytical framework for the rapid characterization of structurally diverse constituents in other complex botanical systems.

## 1 Introduction

Plant-derived natural products remain an important source of chemically diverse molecules with broad applications in health, nutrition, and related industries. In traditional Chinese medicine (TCM), classical herbal prescriptions are widely used in clinical practice, and their therapeutic effects are generally attributed to the integrated actions of multiple constituents rather than to a single active compound (Gong et al., 2020; Ng et al., 2023). Therefore, comprehensive chemical characterization of plant-based formulations is essential for elucidating their material basis, supporting quality evaluation, and facilitating further pharmacological investigation.

However, systematic phytochemical analysis of complex herbal formulations remains challenging. Such matrices usually contain structurally diverse constituents, including flavonoids, phenolic acids, coumarins, terpenoids, alkaloids, amides, and other metabolites, distributed over wide ranges of polarity and abundance (Wang et al., 2026; Zhang and Park, 2025). In addition, the frequent occurrence of structurally related analogues, positional isomers, and low-abundance constituents greatly increases the difficulty of accurate annotation (Çiçek et al., 2024). Structural isomers and trace-level constituents, which may play important roles in bioactivity, further increase analytical difficulty (Yuan et al., 2025). These factors often limit the coverage, efficiency, and confidence of conventional analytical approaches when applied to multi-herb botanical systems.

Liquid chromatography coupled with high-resolution mass spectrometry (LC–HRMS) has become one of the most powerful tools for phytochemical investigation because of its high sensitivity, wide analytical coverage, and accurate mass measurement capability (Arruda et al., 2023; Zhuang et al., 2025). In particular, UPLC-Q Exactive Orbitrap HRMS enables rapid detection of structurally diverse metabolites in complex botanical matrices. Nevertheless, annotation of compounds in herbal systems still depends heavily on manual interpretation of MS/MS fragmentation patterns, which is labor intensive and often insufficient for distinguishing structurally related metabolites and isomers. Although spectral database matching has substantially improved annotation efficiency, database-based identification alone is often inadequate for the systematic recognition of unknown yet structurally related compounds (Wang et al., 2025).

Feature-based molecular networking (FBMN) provides an effective complementary strategy by organizing MS/MS spectra according to spectral similarity, thereby enabling the visualization of molecular families and facilitating the discovery of structurally related metabolites (Nothias et al., 2020; Wang et al., 2016). When integrated with automated feature extraction and multi-source spectral resources, FBMN can improve annotation propagation, assist in the discrimination of isomeric clusters, and enhance the detection of low-abundance analogues in complex plant-derived mixtures. Therefore, a combined strategy involving high-resolution mass spectrometry, multi-database screening, and molecular networking is highly promising for comprehensive phytochemical profiling of botanical formulations.

Xie-Bai-San decoction (XBSD) is a classical plant-based TCM prescription first recorded in Xiao’er Yaozheng Zhijue during the Song Dynasty. It consists of Cortex Mori (the root bark of *Morus alba* L.), Cortex Lycii (the root bark of *Lycium chinense* Mill. or *Lycium barbarum* L.), and Radix et Rhizoma Glycyrrhizae (the root and rhizome of *Glycyrrhiza uralensis* Fisch.), and has long been used to treat cough, wheezing, and other respiratory disorders associated with “lung-heat” syndrome, especially in pediatric patients. Modern studies have also demonstrated its anti-inflammatory and lung-protective activities (Fu, 2016; Tang et al., 2025; Yao et al., 2025; Zhao et al., 2024). Despite its longstanding clinical use and pharmacological relevance, the overall chemical composition of XBSD, especially its structurally related isomers and low-abundance constituents, has not yet been comprehensively characterized.

**FIGURE 1:**
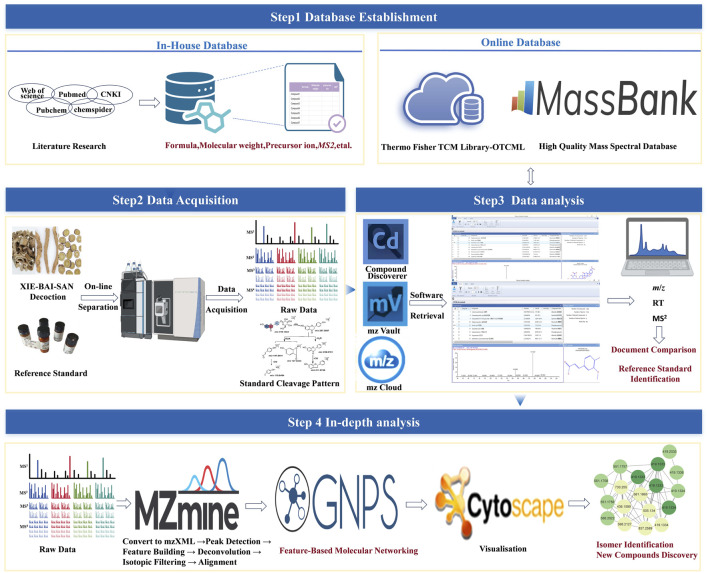
An integrated strategy for chemical characterization of XBSD extract. Some graphical elements were created using BioRender. Created in BioRender. hongjiao, C. (2026) https://BioRender.com/y8aji3x.

In the present study, an integrated analytical strategy was established for the comprehensive phytochemical characterization of XBSD by combining UPLC-Q Exactive Orbitrap HRMS with automated feature extraction, multi-database screening, feature-based molecular networking, and targeted manual validation. As illustrated in Figure 1, this workflow enables systematic structural annotation through the coordinated use of accurate mass measurement, MS/MS fragmentation behavior, spectral matching, and molecular family clustering. Using this strategy, 218 constituents were annotated or tentatively characterized, including 74 compounds reported in XBSD for the first time. In addition, representative MS/MS fragmentation behaviors of major compound classes were summarized to support the annotation of structurally related analogues. This study therefore expands the known chemical profile of XBSD and offers a practical analytical framework for the phytochemical investigation and quality evaluation of other complex plant-derived formulations.

## 2 Materials and methods

### 2.1 Chemicals and reagents

LC–MS grade acetonitrile, methanol, and formic acid were obtained from Thermo Fisher Scientific (United States). Ultrapure water was supplied by Watsons Water. All other chemicals were of analytical grade. All plant materials were purchased from Shouer Pharmaceutical Co., Ltd. (Beijing, China) and authenticated by Professor Jianjun Zhang. Detailed information on all reagents and reference standards is provided in the Supplementary Tables S1, S2.

### 2.2 Sample and reference preparation

XBSD was prepared according to the standard formula and herb-to-water ratio specified by the China Administration of Traditional Chinese Medicine. Cortex Mori (66.5 g), Cortex Lycii (66.5 g), and Radix et Rhizoma Glycyrrhizae (6.8 g) were powdered, sieved (No. 10), soaked in 6.8 L of water for 30 min, and decocted for 30 min to yield approximately 4.1 L of filtrate. The decoction was concentrated under reduced pressure and dried at 60 °C to obtain the XBSD dry extract. For sample preparation, 0.5 g of the extract was refluxed with 25 mL of 50% methanol for 30 min. After cooling and volume adjustment, the solution was diluted 1.5-fold and filtered through a 0.22 μm membrane prior to analysis.

A solution containing a mixture of 13 reference standards was created using methanol, stored at a temperature of 4 °C, and subsequently filtered with a 0.22 μm membrane prior to analysis via UPLC–Q Exactive Orbitrap HRMS.

### 2.3 UPLC–Q Exactive Orbitrap HRMS analysis

XBSD was examined through an Ultimate 3000 UPLC system linked with a Q-Exactive Plus Orbitrap mass spectrometer from Thermo Fisher Scientific, United States.

#### 2.3.1 Liquid chromatographic conditions

Chromatographic separation was performed on an ACQUITY UPLC® HSS T3 column (150 mm × 2.1 mm, 1.8 μm) maintained at 30 °C. The mobile phase consisted of 0.1% formic acid in water (A) and acetonitrile (B). The gradient program was as fol-lows: 0–8 min (2%–7% B), 8–10 min (7%–10% B), 10–12 min (10% B), 12–20 min (10%–13% B), 20–25 min (13%–19% B), 25–27 min (19% B), 27–30 min (19%–27% B), 30–32 min (27% B), 32–40 min (27%–90% B), 40–42 min (90% B), 42–45 min (90%–2% B), and 45–50 min (2% B). The flow rate was 0.2 mL/min, and the injection volume was 2 μL.

#### 2.3.2 Mass spectrometry conditions

Detection via mass spectrometry was carried out employing a heated electrospray ionization source that functioned in both positive and negative ion modes. In the positive ion mode, the electrospray voltage was calibrated to +3.8 kV, while in the negative ion mode, it was adjusted to −3.2 kV. The flow rate of the sheath gas was set to 35 arbitrary units (arb), and the auxiliary gas flow was established at 15 arb. Additionally, the temperature during the process was maintained at 300 °C. For optimal ion transfer, the temperature of the ion transfer tube was held steady at 350 °C. Higher-energy collisional dissociation (HCD) with normalized collision energies set at 20, 40, and 60 eV was utilized to carry out MS/MS fragmentation.

### 2.4 Data acquisition and processing

#### 2.4.1 Construction of the chemical composition database

A local chemical database for XBSD was constructed by systematically collecting reported constituents of Cortex Mori, Cortex Lycii, Radix et Rhizoma Glycyrrhizae, and XBSD from PubMed, Web of Science, and CNKI. Chemical information was supplemented using PubChem and ChemSpider. Compound names, molecular formulas, molecular weights, adduct forms, diagnostic fragment ions, and compound classes were compiled to establish an in-house database.

To expand coverage, the Open Traditional Chinese Medicine Library (OTCML) was incorporated. OTCML provides MS^1^ and MS/MS spectra for constituents derived from more than 1,200 traditional Chinese medicines. Acquisition parameters were aligned with those used in OTCML to ensure spectral comparability. MS/MS spectra were generated using HCD at normalized collision energies of 20, 40, and 60 eV. The MassBank database was additionally used as a reference spectral resource.

#### 2.4.2 Data acquisition

UPLC–Q Exactive Orbitrap HRMS was used for MS/MS acquisition of XBSD samples and reference standards. Spectra of authentic standards were imported into mzVault to establish a local reference library, which was integrated with OTCML and MassBank. By integrating literature evidence, MS/MS spectral analysis, and the fragmentation behaviors of available reference compounds, representative fragmentation features of major compound classes were summarized to assist the annotation of structurally related analogues.

#### 2.4.3 Automated data processing and multi-level compound identification

Raw data were processed in Compound Discoverer (Thermo Fisher Scientific) for peak detection, retention time alignment, background subtraction, and mass recalibration. Retention time (*t*_R_), *m*/*z*, and peak area were extracted for each feature. Molecular formula prediction was performed with a mass tolerance of 5 ppm. Features were filtered using the following criteria: peak area ≥100,000, *t*_R_ between 0.5 and 48.0 min, and exclusion of formulas containing Br, Si, F, as these elements are not naturally present in the constituent herbs.

A multi-level identification workflow was implemented within Compound Discoverer. Experimental MS/MS spectra were first matched against mzCloud, followed by searching a local mzVault library containing reference standard spectra together with curated records from OTCML and MassBank. Candidate structures were further queried in ChemSpider. Structural assignments were refined using characteristic fragment ions combined with MzLogic substructure ranking.

#### 2.4.4 Molecular network construction for identification of unknown compounds and isomers

The raw LC–MS/MS data were initially transformed into the open mzXML format through the use of MSConvert (ProteoWizard). Following this, MZmine 4.4.3 facilitated the detection of chromatographic peaks, alignment of retention times across samples, feature deconvolution, and grouping of isotopes/adducts, which resulted in the creation of a consensus MS/MS spectrum for each identified feature. Ultimately, the quantitative feature table along with the consensus MS/MS spectra were uploaded to the GNPS platform to perform feature-based molecular networking analysis. The parameters for molecular networking were established as follows: a minimum cosine similarity score of 0.7, a minimum of 6 matched fragment ions, and a topK value of 10. Both precursor and fragment ion mass tolerances were configured to 0.02 Da. Using these parameters, a molecular network was constructed in GNPS, and the resultant network was visualized with Cytoscape 3.9.1 (Cytoscape Consortium).

#### 2.4.5 Validation of compound identification results

Preliminary identifications, generated through software-based matching and molecular networking, were further validated in Xcalibur 4.1 by extracting ion chromatograms and performing manual inspection with a 5 ppm mass tolerance window, thereby eliminating false positives.

## 3 Results

### 3.1 Workflow of chemical constituent identification in XBSD

To achieve comprehensive and high confidence characterization of XBSD, a tiered identification workflow integrating automated spectral matching, multi-source database searching, FBMN, and targeted manual validation was established. High-resolution MS and MS/MS data were acquired using UPLC–Q Exactive Orbitrap HRMS and initially processed in Compound Discoverer for feature extraction, formula prediction, and preliminary annotation. Experimental MS/MS spectra were sequentially matched against mzCloud, a locally constructed mzVault library (containing authentic reference standards), and curated entries from OTCML and MassBank. This stepwise matching strategy prioritized high confidence spectral similarity before extending to literature-based candidate retrieval. To further improve annotation coverage and structural discrimination, FBMN was implemented on the GNPS platform. By organizing MS/MS spectra according to fragmentation similarity, molecular networking enabled visualization of structurally related feature clusters and facilitated propagation of structural information from confirmed “seed” compounds to unknown analogues. This approach was particularly advantageous for distinguishing structural isomers and detecting low abundance metabolites that were not confidently resolved by database matching alone. Final structural assignments were established through integrated evaluation of accurate mass measurement (≤5 ppm), MS/MS fragmentation behavior, retention characteristics, molecular networking relationships, and diagnostic fragmentation pathways. This multi-level strategy significantly reduced reliance on manual interpretation while enhancing both annotation confidence and structural consistency across compound classes.

### 3.2 Global chemical profiling and molecular network analysis of XBSD

To improve the reliability and transparency of compound characterization, the annotated constituents were assigned to different confidence levels following the framework of the Metabolomics Standards Initiative (Fiehn et al., 2007; Sumner et al., 2007). Compounds confirmed by authentic reference standards based on retention time (with a tolerance of ±0.05–0.2 min), accurate mass, and MS/MS spectra were classified as level 1 identifications. Compounds matched with literature reports, database records, and diagnostic fragmentation behaviors were considered level 2 annotations, whereas the remaining compounds characterized mainly on the basis of accurate mass, molecular formula prediction, and structural similarity within molecular families were regarded as tentative identifications. Using this analytical framework, a total of 218 chemical constituents were characterized in XBSD. Among them, 13 compounds were unambiguously identified as level 1 by comparison with authentic reference standards, 157 compounds were assigned as level 2 annotations, and 48 compounds were regarded as level 3 tentative identifications. After comparison with previously reported XBSD-related constituents, 74 annotated compounds were considered to be reported in XBSD for the first time, although some of them have been previously reported in individual herbs or other botanical sources. These compounds are marked in Supplementary Table S3. Detailed information for each annotated feature, including retention time, precursor ion, elemental composition, mass deviation, representative fragment ions, and origin of herbs is provided in Supplementary Table S3.

**FIGURE 2:**
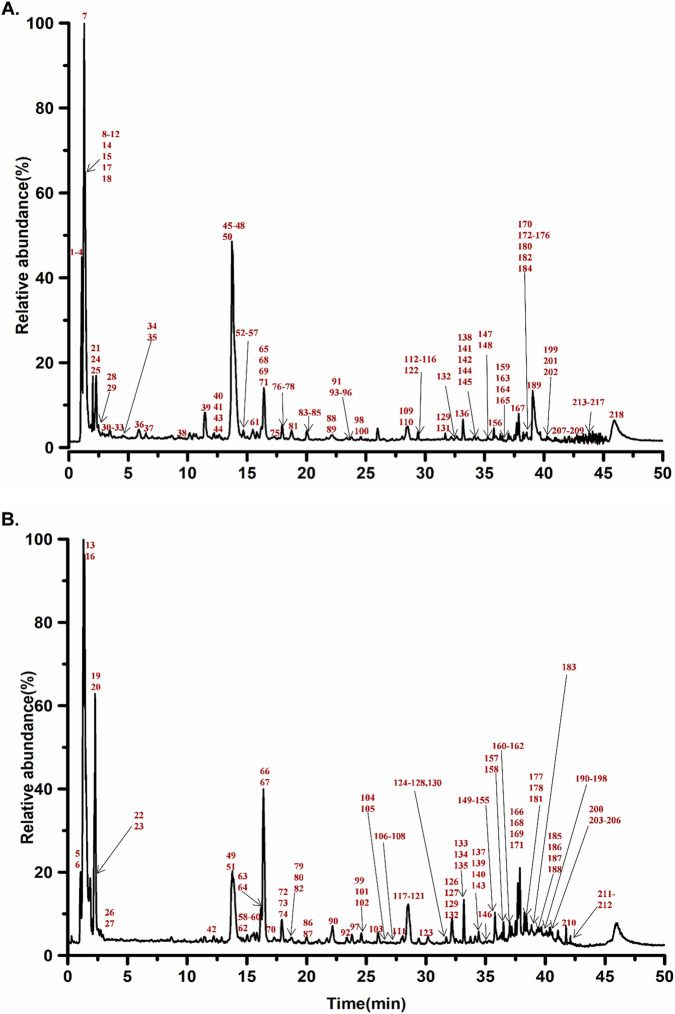
Total ion chromatograms of XBSD extract obtained by UPLC-Q Exactive Orbitrap HRMS in positive **(A)** and negative **(B)** ion modes.

The high-resolution total ion chromatograms acquired in both positive and negative ion modes (Figure 2) revealed the pronounced chemical complexity of XBSD and demonstrated the complementary coverage achieved under the two ionization conditions. Structurally, the chemical profile was dominated by 80 flavonoids, together with 35 organic acids, 23 amides, and 21 terpenoids. In addition, 17 coumarins, 8 saccharides, 6 alkaloids, 5 stilbenes, 3 cyclic peptides, and 20 other minor constituents were detected (Supplementary Figure S1), collectively reflecting the marked chemical and structural diversity of the formulation.

**FIGURE 3:**
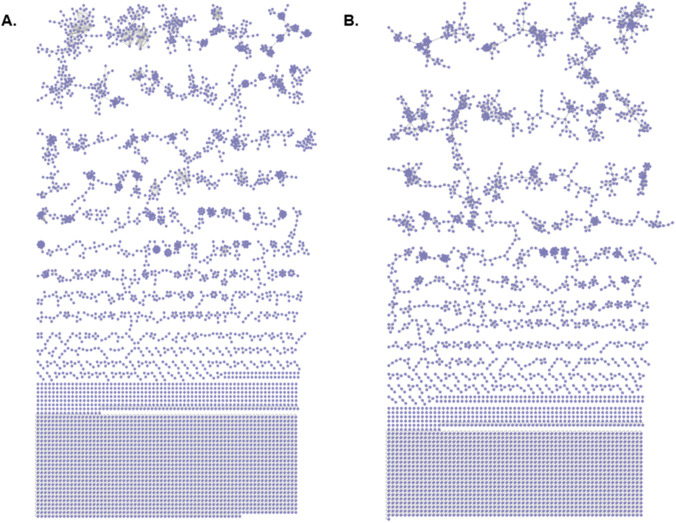
Feature-based molecular networks from MS/MS data acquired in positive **(A)** and negative **(B)** ion modes.

FBMN analysis was independently performed for positive and negative ion modes. Compounds with identical or closely related structures formed discrete subnetworks, enabling visualization of molecular families and facilitating recognition of isomeric and structurally related constituents (Figure 3). All FBMN datasets have been deposited in the GNPS public repository (positive ion mode: https://gnps.ucsd.edu/ProteoSAFe/result.jsp?task=f96391cb49ac472dbf9ede0437f1ab2a&view=view_all_annotations_DB; negative ion mode: https://gnps.ucsd.edu/ProteoSAFe/result.jsp?task=c7a79215c76d48548d6aa71696d998c5&view=view_all_annotations_DB).

### 3.3 Identification of flavonoids

Flavonoids are one of the largest and most diverse groups of plant secondary metabolites, characterized by a C_6_–C_3_–C6 backbone composed of two aromatic rings (A and B) connected by a three-carbon heterocyclic ring (C). They are biosynthesized through the phenylpropanoid pathway from phenylalanine and tyrosine and are widely distributed in plants (Panche et al., 2016). Structural diversity arises from modifications such as hydroxylation, methoxylation, glycosylation, and prenylation, which strongly influence their antioxidant and pharmacological activities (Kumar and Pandey, 2013) total of 80 flavonoid-related constituents were characterized in XBSD. According to structural attributes, these compounds were categorized into O-glycosylated flavonoids, C-glycosides, and their corresponding aglycones. The extensive structural diversity observed within this class highlights the central role of flavonoids in shaping the chemical profile of the formulation.

#### 3.3.1 Flavonoid O-glycosides and related aglycones

Flavonoid O-glycosides are characterized by sugar units attached to the aglycone via C–O linkages. Fragmentation is typically initiated by cleavage of the glycosidic bond. Monoglycosides preferentially lose a hexose unit (162 Da) to yield the aglycone ion, whereas diglycosides undergo stepwise neutral losses of sugar residues. The resulting aglycones further fragment via characteristic neutral losses (e.g., CH_3_, CO, H_2_O) and Retro-Diels–Alder (RDA) cleavage, producing diagnostic ions indicative of flavonoid subclasses.

Peak **118** (*t*_R_ 28.56 min) exhibited a deprotonated ion at *m*/*z* 549.1624 [M-H]^−^ (C_26_H_30_O_13_, 3.90 ppm). MS/MS analysis revealed successive losses of an apiosyl (C_5_H_8_O_4_) and a glucosyl moiety (C_6_H_10_O_5_), generating fragment ions at *m*/*z* 417.1233 and 255.0665. The aglycone ion (*m*/*z* 255.0665) further underwent RDA cleavage to yield *m*/*z* 135.0077 and 119.0490. The ion at *m*/*z* 119.0490 subsequently lost CO to produce *m*/*z* 91.0176. Additionally, a minor rearrangement fragment was observed at *m*/*z* 153.0184. A dehydrated precursor ion at *m*/*z* 531.1040 was also observed. Based on HRMS data and literature comparison (Li et al., 2016; Nie et al., 2024; Zhu et al., 2024), peak **118** was tentatively assigned as liquiritin apioside, and its MS/MS spectrum and proposed fragmentation pathway are shown in Supplementary Figure S2A. Peak **138** (*t*_R_ 34.12 min) displayed a protonated molecular ion at *m*/*z* 431.1335 [M+H]^+^ (C_22_H_22_O_9_, −0.35 ppm). The initial neutral loss of glucose yielded the aglycone ion at *m*/*z* 269.0807, followed by sequential losses of ·CH_3_ and -OH to generate fragment ions at *m*/*z* 254.0572 and 237.0546. Further CO elimination from *m*/*z* 254.0572 generated *m*/*z* 226.0623. An RDA fragment ion at *m*/*z* 137.0238 was observed. Peak **138** was unambiguously confirmed as ononin using an authentic reference standard, and its MS/MS spectrum and proposed fragmentation pathway are shown in Supplementary Figure S2B. Peak **141** (*t*_R_ 34.42 min) showed a protonated ion at *m*/*z* 419.1334 [M+H]^+^ (C_21_H_22_O_9_, −0.30 ppm). After glucose elimination, the aglycone ion at *m*/*z* 257.0807 underwent RDA fragmentation to afford ions at *m*/*z* 147.0441 and 137.0234, with subsequent losses of CO and H_2_O yielding additional fragments. Peak **141** was unequivocally identified as liquiritin through comparison with an authentic reference standard. Its MS/MS spectrum and proposed fragmentation pathway are shown in Supplementary Figure S2C.

**FIGURE 4:**
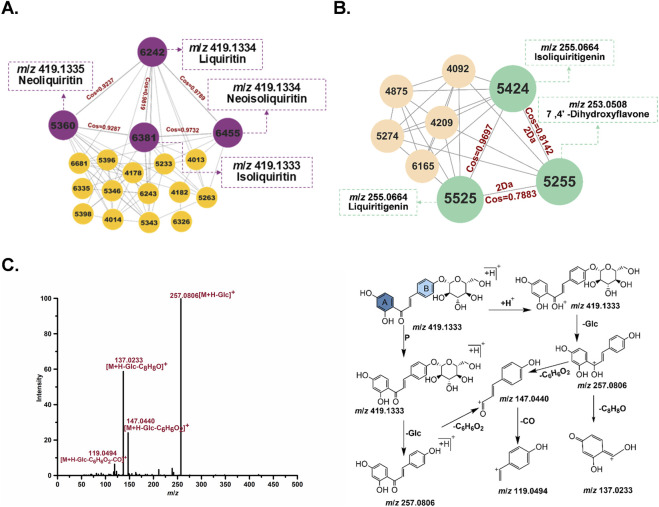
Feature-based molecular networking and proposed fragmentation pathways of flavonoid O-glycosides and related aglycones from XBSD. **(A)** Molecular networking analysis of flavonoid O-glycosides. **(B)** Molecular networking analysis of related aglycones. **(C)** MS/MS spectrum and proposed fragmentation pathways of isoliquiritin.

In the FBMN clustering analysis, nodes #5360, #6455, #6242, and #6381 (peaks **115**, **145**, **141**, and **136**) were grouped into a single family (Figure 4A), all sharing a precursor ion at *m*/*z* 417 [M–H]^−^ but differing in retention times, suggesting isomerism. Node #6242 (peak **141**) was identified as liquiritin based on comparison with an authentic reference standard. Based on GNPS matching, HRMS data, fragmentation patterns, and literature references (Wang et al., 2014), nodes #6381, #5360, and #6455 were tentatively assigned as isoliquiritin, neoisoliquiritin, and neoliquiritin, respectively. Another distinct cluster comprised nodes #5525, #5424, and #5255 (Figure 4B). Nodes #5424 (peak **149**) and #5525 (peak **154**) both exhibited *m*/*z* 255 [M–H]^-^ and were tentatively identified as isoliquiritigenin and liquiritigenin, respectively (Han et al., 2025; Wang et al., 2022). Node #5255 (peak **143**), which differed by 2 Da, was identified as 7,4′-dihydroxyflavone, based on HRMS, MS/MS, and supporting literature (Wang et al., 2022). As an example, node #6381 (peak **136**) displayed a protonated molecular ion at *m*/*z* 419.1333 [M+H]^+^ (C_21_H_22_O_9_, -0.93 ppm). Key fragment ions observed at *m*/*z* 257.0806 [M+H-Glc]^+^, 147.0440 [M+H-Glc-C_6_H_6_O_2_]^+^, 119.0494 [M+H-Glc-C_6_H_6_O_2_-CO]^+^, and 137.0233 [M+H-Glc-C_8_H_8_O]^+^ were consistent with the fragmentation pathway previously described for isoliquiritin (Wang et al., 2014). The corresponding MS/MS spectrum and proposed fragmentation pathway are presented in Figure 4C.

#### 3.3.2 Structural characterization and fragmentation behavior of flavone C-glycosides

**FIGURE 5:**
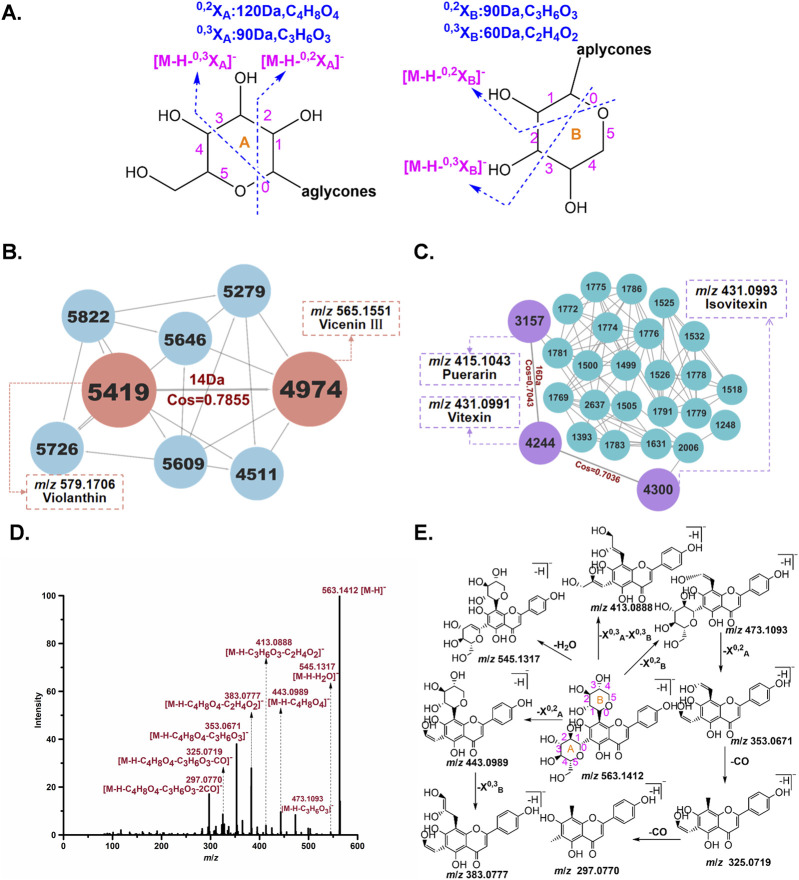
Structural characterization and proposed MS/MS fragmentation pathways of flavone C-glycosides from XBSD. **(A)** Diagnostic neutral losses of hexose- and pentose-substituted C-glycosides in negative ion mode. **(B)** Molecular networking analysis of vicenin-3 and schaftoside. **(C)** Molecular networking analysis of vitexin, isovitexin, and puerarin. **(D)** MS/MS spectrum and proposed fragmentation pathways of vicenin-3.

Seven flavone C-glycosides were characterized in XBSD. Owing to the stability of the C–C glycosidic linkage, direct sugar loss was not observed. Instead, fragmentation was dominated by cross-ring cleavages within the sugar moiety, providing diagnostic neutral losses for sugar identification (Figure 5A). Hexose C-glycosides exhibited typical neutral losses of 60, 90, and 120 Da, generating ions such as [M-H-60]^−^, [M-H-90]^−^, and [M-H-120]^−^. In contrast, pentose C-glycosides mainly showed 60 and 90 Da losses.

In positive ion mode, FBMN revealed several distinct C-glycoside clusters. Nodes #4974 (peak **104**) and #5419 (peak **105**) formed one family (Figure 5B), differing by 14 Da (−CH_2_), and were annotated as vicenin-III and schaftoside based on GNPS matching and literature comparison (Benayad et al., 2014; Frosi et al., 2023; Zhan et al., 2025; Zhao, 2024). Another cluster (Figure 5C) included nodes #4244 (peak **117**) and #4300 (peak **121**), which exhibited highly similar MS/MS spectra and were annotated as vitexin and isovitexin (Zhan et al., 2025), respectively. Node #3157 (peak **86**) displayed a +16 Da shift, consistent with additional hydroxyl substitution, and was annotated as puerarin (Li et al., 2024; Shi et al., 2024).

Peak **104** (*t*_R_ 26.28 min) was selected as a representative example. In negative ion mode, it exhibited a quasimolecular ion at *m*/*z* 563.1412 [M-H]^−^ (C_26_H_28_O_14_, 2.19 ppm). Fragmentation was initiated by a 0,2-cross-ring cleavage of the hexose moiety (A ring), resulting in the loss of C_4_H_8_O_4_ (120 Da) to yield *m*/*z* 443.0989. Subsequent 0,3-cross-ring cleavage of the pentose moiety (B ring) led to loss of C_2_H_4_O_2_ (60 Da), producing *m*/*z* 383.0777. An alternative fragmentation pathway was also observed. In this route, the pentose moiety first underwent 0,2-cross-ring cleavage (−90 Da), generating *m*/*z* 473.1093, followed by 0,2-cross-ring cleavage of the hexose moiety (−120 Da) to afford *m*/*z* 353.0671. This ion further underwent consecutive CO eliminations, yielding fragment ions at *m*/*z* 325.0719 and 297.0770. These fragmentation behaviors were consistent with previously reported data (Benayad et al., 2014; Frosi et al., 2023). Accordingly, Peak **104** was annotated as vicenin-3. The MS/MS spectrum and proposed fragmentation pathway are shown in Figure 5D.

### 3.4 Identification of stilbenes

Stilbenes are a class of plant-derived polyphenols characterized by a 1,2-diphenylethylene (C_6_–C_2_–C_6_) backbone (Teka et al., 2022). The root bark of Morus alba (Cortex Mori) contains stilbenes such as mulberroside A, oxyresveratrol, and their polysaccharide glycosides, which are important active constituents in Moraceae plants. Studies have shown that these stilbenes exhibit significant anti-inflammatory, antioxidant, anti-aging, and glucolipid metabolism-improving activities (Hankittichai et al., 2020; Liu et al., 2023; Liu et al., 2025; Zhu et al., 2026).

Five stilbenes were characterized in XBSD, including oxyresveratrol, mulberroside A, resveratrol, oxyresveratrol-3-O-β-glucoside, and its isomer. In negative ion mode, these compounds exhibited consistent fragmentation pathways. Glycosylated derivatives initially underwent neutral loss of hexose units (−162 Da), generating the corresponding aglycone ions. Subsequent sequential or competitive eliminations of H_2_O (−18 Da), CO (−28 Da), CO2 (−44 Da), and CH_2_O (−30 Da) from the aglycone yielded characteristic stilbene backbone ions at *m*/*z* 225 and 243, which were diagnostic for this subclass.

Peak **70** (*t*_R_ 16.39 min) was selected as a representative compound. It displayed a deprotonated molecular ion [M-H]^−^ at *m*/*z* 567.1725(C_26_H_32_O_14_, 3.03 ppm). In the MS/MS spectrum, the precursor ion successively lost two glucose residues (−162 Da each), affording fragment ions at *m*/*z* 405.1197 [M-H-Glc]^−^ and 243.0663 [M-H-2Glc]^−^. The aglycone ion at *m*/*z* 243.0663 further underwent typical stilbene fragmentation, producing ions at *m*/*z* 225.0556 (−H_2_O), 215.0707 (−CO), and 199.0760 (−CO_2_). These diagnostic fragment ions, together with retention time matching with an authentic reference standard, confirmed peak **70** as mulberroside A. The MS/MS spectrum and proposed fragmentation pathways are shown in Supplementary Figures S3A,B.

### 3.5 Identification of organic acids

Organic acids are low molecular weight metabolites widely present in plant-derived foods, contributing to sensory quality, nutritional value, microbial stability, and shelf life (Sorathiya et al., 2025). In addition, their compositional profiles are increasingly used as chemotaxonomic markers and indicators for quality control and product authentication (Gawenda- Kempczyńska et al., 2022; Seraglio et al., 2021). A total of 35 organic acids were characterized in XBSD, including aliphatic acids, quinic acid derivatives, amino acids, phenolic acids, and one aromatic acid. Among these, chlorogenic acid (peak **64**) and caffeic acid (peak **79**) were unequivocally identified through direct comparison with authentic reference standards.

**FIGURE 6:**
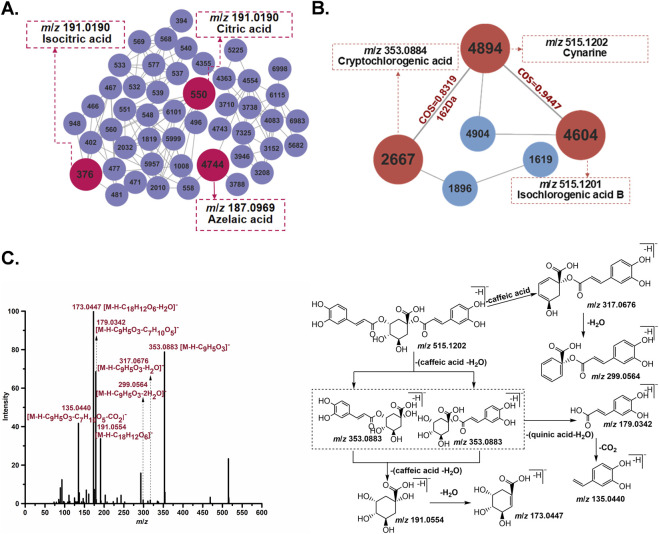
Feature-based molecular networking and proposed MS/MS fragmentation pathways of organic acids from XBSD. **(A)** Molecular networking analysis of citric acid, isocitric acid, and azelaic acid. **(B)** Molecular networking analysis of 1,5-dicaffeoylquinic acid, cryptochlorogenic acid, and isochlorogenic acid B. **(C)** MS/MS spectrum and proposed fragmentation pathways of 1,5-dicaffeoylquinic acid.

FBMN analysis revealed several molecular families within the organic acid class. Nodes #550 (peak **23**) and #376 (peak **19**) shared identical precursor ions and highly similar MS/MS spectra, supporting an isomeric relationship, whereas node #4744 (peak **127**) differed by 4 Da. Integrated interpretation of GNPS matching results, high-resolution mass data, and fragmentation behavior enabled the annotation of these nodes as citric acid, isocitric acid, and Azelaic Acid, respectively (Figure 6A) (Chen et al., 2019; Xu et al., 2022; Zhao, 2024). Another cluster (Figure 6B) consisted of nodes #4894 (peak **133**), #4604 (peak **124**), and #2667 (peak **74**). Nodes #4894 and #4604 exhibited the same precursor ion at *m*/*z* 515 and nearly identical MS/MS spectra, indicating isomeric dicaffeoylquinic acids, while node #2667 (peak **74**) showed a 162 Da mass increase consistent with glucosyl substitution. Accordingly, these nodes were annotated as cynarine, isochlorogenic acid B, and cryptochlorogenic acid, respectively.

Peak **133** (*t*_R_ 32.85 min) displayed a deprotonated molecular ion at *m*/*z* 515.1202 [M-H]^−^ (C_25_H_24_O_12_, 3.58 ppm). Under HCD conditions, prominent fragment ions at *m*/*z* 353.0883, 173.0447, and 135.0440 were observed, corresponding to stepwise cleavage of caffeoyl and quinic acid moieties. These fragmentation characteristics were consistent with previously reported data for cynarine (Petrova et al., 2025). The MS/MS spectrum and proposed fragmentation pathway are presented in Figure 6C.

In general, the organic acids in XBSD are fragmented primarily via the cleavage of ester or glycosidic bonds. This process produced characteristic neutral losses of caffeoyl (162 Da), feruloyl (176 Da), glucosyl (162 Da), H_2_O (18 Da), or CO_2_ (44 Da), providing a robust basis for structural elucidation.

### 3.6 Identification of terpenoids

Modern pharmacological studies have shown that triterpenoids possess significant potential in anti-inflammatory, antitumor, metabolic diseases, and organ protection (Malik and Mandal, 2024; Miranda et al., 2022). In this study, 21 terpenoids were characterized in XBSD, including 19 triterpenoids, 1 sesquiterpenoid, and 1 monoterpenoid. In MS analysis, these compounds were primarily characterized by sequential neutral losses of glucuronic acid moieties and dehydration of the aglycone.

**FIGURE 7:**
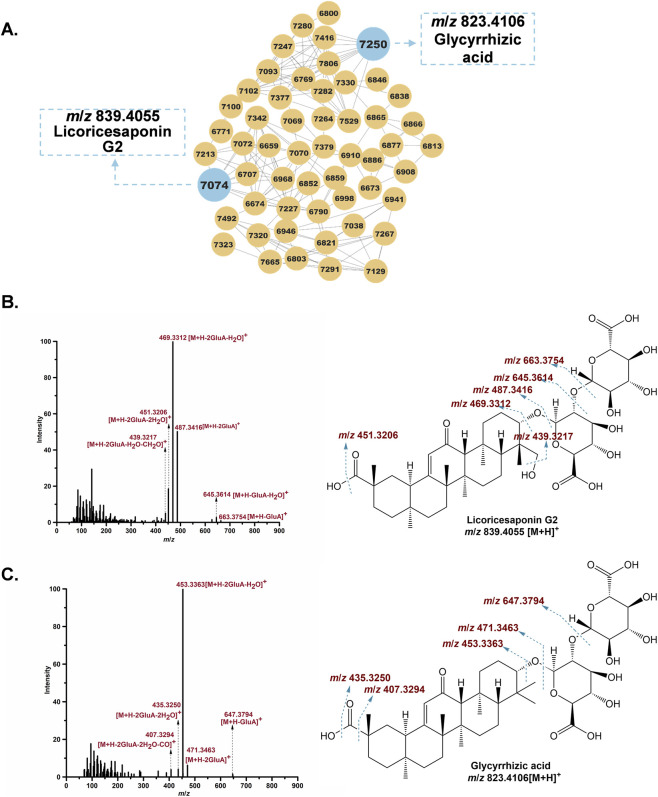
Feature-based molecular networking and proposed fragmentation pathways of triterpenoids from XBSD. **(A)** Molecular network analysis of glycyrrhizin G_2_ and glycyrrhizic acid. **(B)** MS/MS spectrum and proposed fragmentation pathways of glycyrrhizic acid. **(C)** MS/MS spectrum and proposed fragmentation pathways of glycyrrhizin G2.

Peaks **165** (*t*_R_ 37.51 min) and **167** (*t*_R_ 37.87 min) were selected as representative examples. In the FBMN, they clustered within the same molecular family (Figure 7A), with precursor ions differing by 16 Da, corresponding to one additional oxygen atom. GNPS matching tentatively assigned peaks **165** and **167** as glycyrrhizin G_2_ and glycyrrhizic acid, respectively. In positive ion mode, peak **165** displayed a protonated molecular ion at *m*/*z* 839.4055 [M+H]^+^ (C_42_H_62_O_17_), whereas peak **167** showed *m*/*z* 823.4106 [M+H]^+^ (C_42_H_62_O_16_). Under HCD conditions, both compounds exhibited analogous fragmentation pathways. Initial neutral loss of one glucuronic acid unit (−176 Da) generated fragment ions at *m*/*z* 663.3754 (peak **165**) and 647.3794 (peak **167**), followed by elimination of a second glucuronic acid (−352 Da) to yield ions at *m*/*z* 487.3416 and 471.3463, respectively. Subsequent fragmentation patterns diverged. For peak **167**, the ion at *m*/*z* 471.3463 underwent successive dehydration to form *m*/*z* 453.3363 and 435.3250, followed by CO loss to produce *m*/*z* 407.3294. In contrast, the ion at *m*/*z* 487.3416 from peak **165** predominantly underwent dehydration (*m*/*z* 469.3312 and 451.3206) and subsequent CH_2_O loss to yield *m*/*z* 439.3217. Additionally, the intermediate ion at *m*/*z* 663.3754 (peak **165**) further dehydrated to *m*/*z* 645.3614. Peak **167** was unequivocally identified as glycyrrhizic acid by comparison with an authentic reference standard, whereas peak **165** was annotated as glycyrrhizin G2 based on MS/MS fragmentation features and literature data (Avula et al., 2022). Their MS/MS spectra and proposed fragmentation pathways are presented in Figures 7B,C, respectively.

### 3.7 Identification of amides

Plant amides are nitrogen-containing specialized metabolites formed through the N-acylation of biogenic amines with hydroxycinnamic or fatty acid moieties (Bassard et al., 2010). In the present study, the amide constituents of XBSD were systematically characterized. A total of 23 amides were identified, including 10 spermidine-type, 4 putrescine-type, 2 cadaverine-type, 5 other phenolamides, and 2 nonphenolic amides.

FBMN analysis clustered nodes #6439 (peak **142**), #2249 (peak **39**), and #6518 (peak **147**) within the same molecular family (Supplementary Figure S4A). GNPS annotation assigned these nodes to N-coumaroyltyramine (E)-N-caffeoylputrescine, and N-feruloyltyramine, respectively, and the assignments were supported by HRMS measurements and MS/MS fragmentation data (Wang et al., 2017; Zhang et al., 2014; Zhao, 2024). Peak **39** (*t*_R_ 11.42 min) and exhibited a protonated molecular ion [M+H]^+^at *m*/*z* 251.1388 (C_13_H_18_N_2_O_3_, −0.83 ppm) in positive ion mode. The MS/MS spectrum showed fragment ions at *m*/*z* 234.1123 corresponding to NH_3_ loss and at *m*/*z* 163.0389 generated by cleavage of the C_4_H_12_N_2_ moiety. Additional fragment ions at *m*/*z* 89.1079 and 72.0815 were detected, corresponding to protonated putrescine and its subsequent NH_3_ loss, respectively. The MS/MS spectrum and proposed fragmentation pathways are presented in Supplementary Figure S4B. Peak **147** (*t*_R_ 35.40 min) and displayed an [M+H]^+^ ion at *m*/*z* 314.1385 (C_18_H_19_NO_4_, −0.72 ppm). Its MS/MS spectrum was characterized by a fragment ion at *m*/*z* 177.0546 formed after neutral loss of C_8_H_11_NO (137 Da). Subsequent fragment ions were observed at *m*/*z* 149.0602 and 121.0650 following sequential CO losses, as well as at *m*/*z* 145.0284 corresponding to CH_3_OH loss. The MS/MS spectrum and proposed fragmentation pathways are shown in Supplementary Figure S4C.

For spermidine- and spermine-type phenolamides, fragmentation was characterized by initial amide bond cleavage followed by stepwise degradation of the polyamine backbone (Li et al., 2014; Li et al., 2018). Peak **52** (*t*_R_ 14.07 min) showed an [M+H]^+^ ion at *m*/*z* 531.3175 (C_28_H_42_N_4_O_6_, −0.40 ppm). In the MS/MS spectrum, the precursor ion underwent a loss of C_12_H_15_NO_3_ (221 Da), followed by sequential losses of 17 Da and 71 Da, yielding fragment ions at *m*/*z* 293.1858 [M+H-C_12_H_15_NO_3_-NH_3_]^+^ and *m*/*z* 222.1125 [M+H-C_12_H_15_NO_3_-NH_3_-C_4_H_9_N]^+^, respectively. Additional fragment ions were observed at *m*/*z* 513.3055 [M+H-H_2_O]^+^, 165.0547 [M+H-C_19_H_34_N_4_O_3_]^+^, and 123.0442 [M+H-C_19_H_34_N_4_O_3_-CO-CH_2_]^+^. Peak 52 was unequivocally identified as kukoamine B by comparison with an authentic reference standard.

### 3.8 Identification of coumarins

Coumarin derivatives exhibit a broad spectrum of pharmacological activities, including anti-inflammatory, antitumor, antioxidant, antiinfective, and neuroregulatory effects. Moreover, structural modification serves as an effective strategy to fine-tune their target affinity and biological activity (Garg et al., 2020). Seventeen coumarins were annotated in XBSD. In MS analysis, these compounds typically exhibit initial neutral losses of substituents, such as glycosidic cleavage (−162 Da) or demethylation (−15 Da), followed by characteristic stepwise losses of CO (28 Da) and CO_2_ (44 Da) from the coumarin core, providing key diagnostic information.

Peaks **73** and **106** were selected as representative examples. Peak **73** (*t*_R_ 17.01 min) showed a [M–H]^−^ ion at *m*/*z* 177.0186 (C_9_H_6_O_4_, 1.89 ppm). Its MS/MS spectrum displayed fragment ions at *m*/*z* 149.0233 [M-H-CO]^−^, 133.0283 [M-H-CO_2_]^−^, 105.0333 [M-H-CO_2_-CO]^−^, and 89.0383 [M-H-2CO_2_]^−^, consistent with successive CO and CO_2_ losses from the coumarin nucleus. Comparison with an authentic standard confirmed peak **73** as esculetin, and its MS/MS spectrum and proposed fragmentation pathways are shown in Supplementary Figure S5A. Peak **106** (*t*_R_ 26.47 min) exhibited a [M–H]^−^ ion at *m*/*z* 191.0344 (C_10_H_8_O_4_, 2.59 ppm). Its MS/MS spectrum showed characteristic ions at *m*/*z* 176.0107 [M-H-CH_3_]^−^, 148.0156 [M-H-CH_3_-CO]^−^, and 104.0254 [M-H-CH_3_-CO-CO_2_]^−^. Based on its fragmentation pattern and authentic standard, peak **106** was tentatively assigned as scopoletin, and its MS/MS spectrum and proposed fragmentation pathways are shown in Supplementary Figure S5B.

### 3.9 Identification of saccharides

The identification of saccharides in complex natural products is challenging due to their high structural similarity and isomeric diversity. In this study, FBMN was employed to systematically characterize eight saccharides in XBSD. FBMN analysis revealed that nodes #272 (peak **18**), #215 (peak **11**), #204 (peak **8**), #249 (peak **17**), #208 (peak **10**), and #207 (peak **9**) were grouped into the same molecular family. This clustering suggested highly similar precursor ions and MS/MS fragmentation patterns (Supplementary Figure S6).

Peak **18** (*t*_R_ 1.41 min) displayed a dominant [M+H-H_2_O]^+^ ion at *m*/*z* 325.1125 (C_12_H_22_O_11_, −0.40 ppm). The corresponding MS/MS spectrum showed characteristic fragment ions at *m*/*z* 163.0603 [M+H-H_2_O-Glc]^+^, 145.0494 [M+H-Glc-2H_2_O]^+^, 127.0391 [M+H-Glc-3H_2_O]^+^, and 85.0290 [M+H-Glc-3H_2_O-CO_2_]^+^. Based on the diagnostic fragmentation behavior and comparison with literature data (Xu et al., 2022; Zhao, 2024), peak **18** was assigned as sucrose. Peak **17** exhibited a precursor ion at *m*/*z* 343.1232 [M+H]^+^, while peak **8** (node #204) showed a precursor ion at *m*/*z* 325.1124 [M+H-H_2_O]^+^. Both peaks shared the molecular formula C_12_H_22_O_11_ and presented highly similar MS/MS spectra to sucrose. Based on GNPS spectral matching and fragmentation characteristics, peak **17** was tentatively identified as lactulose, and peak 8 was assigned as D-galaturonate. Peak **9** (*t*_R_ 1.32 min) showed a [M+H-H_2_O]^+^ion at *m*/*z* 487.1653 (C_18_H_32_O_16_), while peak **10** (*t*_R_ 1.32 min) exhibited a [M+H-H_2_O]^+^ ion at *m*/*z* 649.2180 (C_24_H_42_O_21_). The 162 Da mass difference between these ions indicated the addition of one hexose residue, suggesting a difference of one glucose unit in polymerization degree. Their sequential neutral losses and typical glycosidic cleavage fragments supported the assignment of peak **9** as maltotriose and peak **10** as maltotetraose. Peak **11** (*t*_R_ 1.34 min) displayed a [M+NH_4_]^+^ ion at *m*/*z* 360.1496 and was annotated as D-trehalose based on accurate mass. All saccharides were assigned with mass errors within 5 ppm.

In positive ion mode, the saccharides exhibited consistent fragmentation pathways, characterized by initial dehydration to form [M+H-H_2_O]^+^ ions, followed by 0,2A^−^, 2,4A^−^, and B-type cleavages of glucose units. These processes generated predominant fragment ions at *m*/*z* 145 (C_6_H_9_O_4_^+^), 127 (C_6_H_7_O_3_^+^), and 85 (C_4_H_5_O_2_^+^), which explained their clustering within a single molecular family in the FBMN analysis.

### 3.10 Identification of alkaloids

Six alkaloids were characterized in XBSD, including N-quaternary ammonium alkaloids (betaine, trigonelline, and N-methylgastrodine), an aliphatic amine alkaloid (histamine), a polyhydroxy alkaloid (1-deoxynojirimycin), and an aromatic hydroxy alkaloid (2-hydroxyquinoline). Peaks **14** and **3** were selected as representative compounds to illustrate the fragmentation behavior of different alkaloid subclasses.

Peak **14** (*t*_R_ 1.37 min) exhibited a protonated molecular ion [M+H]^+^ at *m*/*z* 138.0549 (C_7_H_7_NO_2_, −0.40 ppm). The MS/MS spectrum displayed characteristic fragmentation of N-quaternary ammonium alkaloids. Direct decarboxylation (−44 Da) generated the base peak at *m*/*z* 94.0656 (C_6_H_8_N^+^), while subsequent CO loss (−28 Da) produced an ion at *m*/*z* 110.0603 (C_6_H_7_NO^+^). Further elimination of HCN (−27 Da) from the base peak yielded *m*/*z* 67.0550. These diagnostic fragmentations are consistent with the reported MS behavior of trigonelline, leading to its identification. The MS/MS spectrum and proposed fragmentation pathways are presented in Supplementary Figure S7A. Peak **3** (*t*_R_ 1.12 min) showed an [M+H]^+^ ion at *m*/*z* 164.0917 (C_6_H_13_NO_4_, −0.15 ppm). Its MS/MS spectrum was characterized by sequential dehydration, a typical fragmentation pathway of polyhydroxy alkaloids, producing ions at *m*/*z* 146.0813 [M+H-H_2_O]^+^, 128.0708 [M+H-2H_2_O]^+^, and 110.0604 [M+H-3H_2_O]^+^. This stepwise loss of water molecules is characteristic of multiple hydroxyl substitutions and supported its assignment as 1-deoxynojirimycin (Shi et al., 2024; Xu et al., 2022; Zhao, 2024). The corresponding MS/MS spectrum and proposed fragmentation pathways are shown in Supplementary Figure S7B.

### 3.11 Identification of cyclic peptides

Cyclic peptides, characterized by their head-to-tail cyclized backbone, exhibit enhanced metabolic stability and diverse bioactivities compared to their linear counterparts (Martian et al., 2025). Three cyclic peptides were characterized in XBSD, namely, lyciumin B, lyciumin A, and lyciumin D. In ESI-MS, these compounds exhibited consistent diagnostic fragmentation, characterized by amide bond cleavage, neutral loss of formaldehyde (−30 Da) from threonine residues in the cyclic tetrapeptide backbone, and characteristic loss of proline dipeptide units (−208 Da).

**FIGURE 8:**
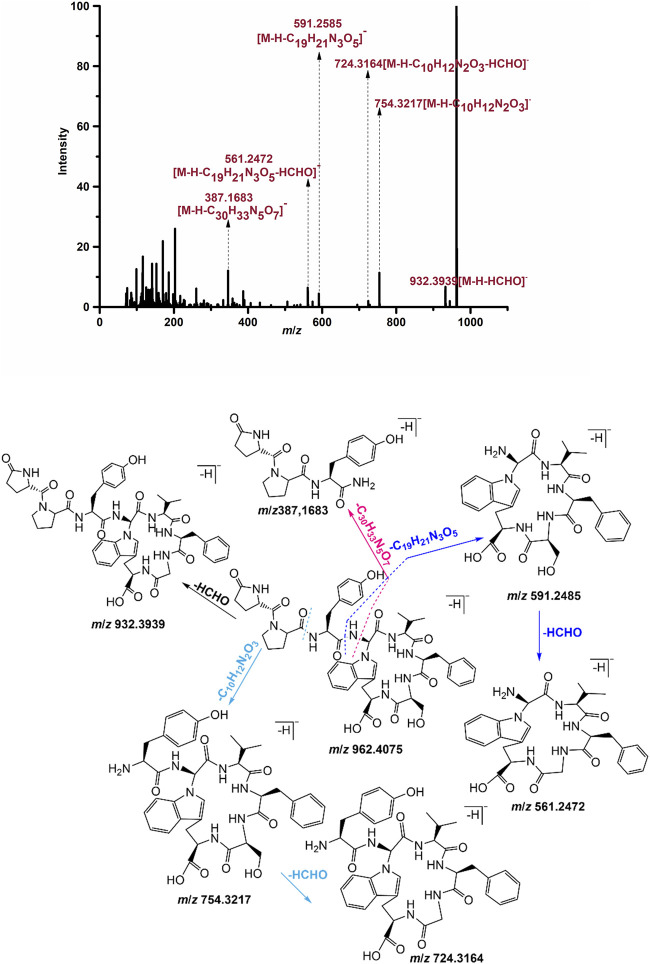
MS/MS spectrum and proposed fragmentation pathways of lyciumin D from XBSD.

Peak **155** was selected as a representative example. It showed a [M-H]^−^ ion at *m*/*z* 962.4075 (C_49_H_57_N_9_O_12_, 3.18 ppm) with a retention time of 36.34 min. Its MS/MS fragmentation proceeded mainly via two pathways. First, the precursor ion lost 208 Da to yield *m*/*z* 754.3217, followed by further loss of HCHO (−30 Da) to generate *m*/*z* 724.3164; alternatively, direct loss of HCHO produced *m*/*z* 932.3939. Second, amide bond cleavage between the cyclic tetrapeptide core and its substituent generated complementary ions at *m*/*z* 591.2485 and 387.1683, with the former further losing 30 Da to form *m*/*z* 561.2472. These fragmentation features were consistent with reported MS behavior of lyciumin D (Jiang et al., 2020; Wang et al., 2024). Based on its retention time and HRMS data, peak **155** was assigned as lyciumin D. The corresponding MS/MS spectrum and proposed fragmentation pathways are shown in Figure 8.

## 4 Discussion

The present study revealed a chemically diverse constituent profile of XBSD, dominated by flavonoids, followed by organic acids, amides, and terpenoids. This distribution reflects the phytochemical contributions of its component herbs, including flavonoids and stilbenoids from Cortex Mori, phenolic amides and cyclic peptides from Cortex Lycii, and liquiritin-type flavonoids and triterpenoid saponins from Radix et Rhizoma Glycyrrhizae. Thus, the characterized profile provides useful herb-origin traceability for understanding the material basis of XBSD.

From an analytical perspective, the coexistence of numerous glycosides, structurally related analogues, and positional isomers remains challenging for reliable annotation. The novelty of this work lies not in the use of LC-HRMS or FBMN alone, but in the integration of authentic standards, an in-house database, TCM-related databases, literature-assisted MS/MS interpretation, and FBMN-based molecular family analysis into a confidence-level-based annotation workflow for XBSD. This strategy facilitated the propagation of structural information from confirmed “seed” compounds to unknown analogues and isomers, improving the annotation of structurally related constituents that are difficult to resolve by database searching alone.

Using this workflow, 218 constituents were characterized in XBSD, including 74 compounds reported in this formula for the first time. To improve the reliability and transparency of qualitative annotation, XBSD sample preparation consistency was evaluated by HPLC quantification of major marker compounds before HRMS analysis. In addition, features were filtered using a peak area threshold of ≥100,000 and mass accuracy within 5 ppm, and all annotations were manually checked based on MS/MS fragmentation patterns in Xcalibur. The annotated constituents were further assigned to different confidence levels following the MSI framework.

Nevertheless, this study remains a qualitative phytochemical profiling study rather than a quantitative metabolomics investigation. Analytical replicate injections and pooled QC samples were not included; therefore, feature-level reproducibility parameters such as peak-area RSDs were not calculated. In addition, although the 50-min UPLC gradient helped ensure broad chemical coverage, acceptable peak shape, and sufficient MS/MS acquisition for low-abundance or isomeric compounds, it was not optimized for high-throughput analysis. Future work should incorporate replicate injections, pooled QC samples, expanded authentic standards, quantitative validation, and further gradient optimization to improve analytical reproducibility, structural resolution, and throughput.

## 5 Conclusion

In summary, this study provided a systematic chemical characterization of XBSD and expanded current knowledge of its constituent composition. The results offer an important chemical basis for future studies on its pharmacological activity and quality evaluation. In addition, this work demonstrates the practical value of combining spectral resources with network-based analysis in the study of traditional herbal formulas, and offers a scalable framework for the chemical investigation of other complex botanical preparations.

## Data Availability

The original contributions presented in the study are publicly available. This data can be found here: 10.6084/m9.figshare.32628078.
