# Supplementary material for: An integrated annotation strategy for the phytochemical characterization of Xie-Bai-San decoction based on UPLC-Q Exactive Orbitrap HRMS, multi-database screening, and feature-based molecular networking
Source: Front Chem. 2026 Jun 17;14:1848728. doi: 10.3389/fchem.2026.1848728 (PMC13318910; doi:10.3389/fchem.2026.1848728)
Supplement: Supplementary file 1 [file DataSheet1.docx]

Supplementary Material

# Supplementary Tables

**Table S1.** Detailed information of the 13 reference standards.

| **Reference Standard** | **Manufacturer** | **Batch Number** | **Purity** |
| --- | --- | --- | --- |
| Mulberroside A | Yuanye Biotechnology Co., Ltd. (Shanghai, China) | J10GB151388 | ≥ 98% |
| Kukoamine B |  | JB271819 | ≥ 98% |
| Ammonium glycyrrhizinate |  | M04GB140062 | ≥ 98% |
| Liquiritin |  | J29IB221288 | ≥ 98% |
| Caffeic acid |  | W16O10B100366 | ≥ 98% |
| Esculetin |  | C25J7Y18390 | ≥ 98% |
| cis-4-coumaric acid |  | SN1111GA14 | ≥ 98% |
| Rutin |  | JO1IB203749 | ≥ 98% |
| Chlorogenic acid | National Institutes for Food and Drug Control (Beijing, China) | 110753-201817 | 96.80% |
| Scopoletin | Nanjing Dilger Medical Technology Co., Ltd. (Nanjing, China) | S03690 | ≥ 98% |
| Hyperoside |  | Y04A9X62302 | ≥ 98% |
| Ononin | Chengdu Pufeide Biotechnology Co., Ltd. (Chengdu, China) | 130507 | ≥ 98% |
| Formononetin | Chengdu Pusi Biotechnology Co., Ltd. (Chengdu, China) | PS000674 | ≥ 98% |

**Table S2.** Plant materials used in this study.

| **Plant Materials** | **Batch number** |
| --- | --- |
| *Morus alba* L*.*  [Moraceae; Mori cortex] | 230301 |
| *Lycium chinense* Mill*.*  [Solanaceae; Lycii cortex] | 2411033 |
| *Glycyrrhiza uralensis* Fisch.  [Fabaceae; Glycyrrhizae Radix Et Rhizoma] | 2406035 |

**Table S3.** Identification of chemical compounds of XBSD.

| **Peak No.** | ***t*_R_**  **(min)** | **Precursor ion** | **Observed mass**  **(Da)** | **Molecular formula** | **Error**  **(ppm)** | **Major fragments** | **Proposed compounds** | **Compound**  **classification** | **Origin of**  **herbs** |
| --- | --- | --- | --- | --- | --- | --- | --- | --- | --- |
| 1 | 1.07 | [M+H]^+^ | 177.1386 | C_11_H_16_N_2_ | -0.26 | 98.9757,84.9602 | N-Methyl Anabasine^#^ | A | CL |
| 2 | 1.11 | [M+H]^+^ | 112.0872 | C_5_H_9_N_3_ | 2.82 | 95.0609,68.0502 | Histamine^#^ | A | CL |
| 3 | 1.12 | [M+H]^+^ | 164.0917 | C_6_H_13_NO_4_ | -0.15 | 146.0813,128.0708,110.0604,82.0658,69.0343 | 1-Deoxynojirimycin^#^ | A | CM |
| 4 | 1.21 | [M+H]^+^ | 175.1189 | C_6_H_14_N_4_O_2_ | -0.47 | 158.0926,116.0709,70.0659 | DL-Arginine^#^ | B | CM/CL/RRG |
| 5 | 1.27 | [M-H]^-^ | 195.0503 | C_6_H_12_O_7_ | 2.11 | 177.0396,159.0289 | D-Gluconic Acid^#^ | B | CM/RRG |
| 6 | 1.27 | [M-H]^-^ | 209.0298 | C_6_H_10_O_8_ | 3.00 | 133.0131,85.0281 | Mucic Acid^#^ | B | CM/CL |
| 7 | 1.29 | [M+H]^+^ | 118.0864 | C_5_H_11_NO_2_ | 1.23 | 59.0738,58.0660 | Betaine^#^ | A | CM/CL |
| ^√^8 | 1.32 | [M+H-H_2_O]^+^ | 325.1124 | C_12_H_22_O_11_ | -1.61 | 145.0496,127.0391,85.0291 | D-galaturonate^*^ | C | CM/CL/RRG |
| ^√^9 | 1.32 | [M+H-H_2_O]^+^ | 487.1653 | C_18_H_32_O_16_ | -0.47 | 145.0495,127.0391,85.0290 | Maltotriose^*^ | C | CM/CL/RRG |
| ^√^10 | 1.32 | [M+H-H_2_O]⁺ | 649.2180 | C_24_H_42_O_21_ | -0.85 | 325.1127,145.0495,85.0290 | α-Maltotetraose^#^ | C | CM/CL/RRG |
| ^√^11 | 1.34 | [M+NH_4_]^+^ | 360.1496 | C_12_H_22_O_11_ | -1.21 | 163.0600,145.0496,127.0391,85.0290 | D-Trehalose^*^ | C | CM/CL/RRG |
| ^√^12 | 1.34 | [M+H]^+^ | 266.1232 | C_10_H_19_NO_7_ | -0.86 | 248.1127,230.1022,98.0604 | D-1-[(3-Carboxypropyl) Amino]-1-Deoxyfructose^*^ | C | CM/CL/RRG |
| 13 | 1.36 | [M-H]^-^ | 179.0553 | C_6_H_12_O_6_ | 1.43 | 161.0446,143.0336,89.0230,71.0124 | Fructose^#^ | C | CM |
| 14 | 1.37 | [M+H]^+^ | 138.0549 | C_7_H_7_NO_2_ | -0.40 | 110.0603,94.0656,67.0550 | Trigonelline^*^ | A | CM/RRG |
| 15 | 1.37 | [M+H]^+^ | 116.0708 | C_5_H_9_NO_2_ | 1.77 | 98.0969,70.0659 | Proline^#^ | B | CL |
| 16 | 1.39 | [M-H]^-^ | 191.0553 | C_7_H_12_O_6_ | 1.34 | 173.0448,147.0287,85.0281 | Quinic Acid^#^ | B | CM/CL |
| ^√^17 | 1.40 | [M+H]^+^ | 343.1232 | C_12_H_22_O_11_ | -0.32 | 163.0603,145.0494,127.0391,85.0290 | Lactulose^*^ | C | CM/CL/RRG |
| 18 | 1.41 | [M+H-H_2_O]⁺ | 325.1125 | C_12_H_22_O_11_ | -0.40 | 163.0603,145.0494,127.0391,85.0290 | Sucrose^#^ | C | CM/RRG |
| ^√^19 | 1.50 | [M-H]^-^ | 191.0190 | C_6_H_8_O_7_ | 2.00 | 173.0084,154.9977,129.0182,111.0075 | Isocitric Acid^#^ | B | CL |
| 20 | 1.52 | [M-H]^-^ | 133.0131 | C_4_H_6_O_5_ | -0.75 | 115.0024,89.0230,71.0124 | (±)-Malic Acid^#^ | B | CM/CL |
| ^√^21 | 2.01 | [M+H]^+^ | 203.2229 | C_10_H_26_N_4_ | -0.51 | 129.1387,112.1124 | Spermine^*^ | D | CL |
| ^√^22 | 2.28 | [M-H]^-^ | 147.0288 | C_5_H_8_O_5_ | 0.14 | 129.0181,102.9475,61.9869 | 3-Hydroxyglutaric Acid* | B | CL |
| 23 | 2.29 | [M-H]^-^ | 191.0190 | C_6_H_8_O_7_ | 2.10 | 173.0083,129.0181,111.0075 | Citric Acid^#^ | B | CM/CL/RRG |
| ^√^24 | 2.34 | [M+H-H_2_O]^+^ | 259.1285 | C_11_H_20_N_2_O_6_ | -1.19 | 213.1234,195.1128,130.0500,84.0814 | L-Saccharopine^*^ | B | CL |
| ^√^25 | 2.35 | [M+H]^+^ | 364.0645 | C_10_H_14_N_5_O_8_P | 0.09 | 152.0567,135.0300,85.0290 | Guanosine Monophosphate^*^ | D | CM/CL/RRG |
| 26 | 2.54 | [M-H]^-^ | 188.0558 | C_7_H_11_NO_5_ | -0.52 | 170.0451,144.0656,128.0341,102.0547 | N-Acetyl-L-Glutamic Acid^#^ | B | CM |
| ^√^27 | 2.58 | [M-H]^-^ | 243.0623 | C_9_H_12_N_2_O_6_ | 4.56 | 200.0559,174.9553,110.0235 | Arabinosyl-Uracil^*^ | D | CM/CL/RRG |
| 28 | 2.71 | [M+H]^+^ | 346.0545 | C_10_H_12_N_5_O_7_P | -0.18 | 152.05675,135.03014 | Cyclic GMP^*^ | D | CM |
| 29 | 2.97 | [M+H]^+^ | 182.0812 | C_9_H_11_NO_3_ | 0.06 | 165.0546,136.0757 | L-Tyrosine^#^ | B | CL/RRG |
| 30 | 3.16 | [M+H]^+^ | 132.1020 | C_6_H_13_NO_2_ | 0.64 | 86.097 | L-Isoleucine^#^ | B | CL/RRG |
| 31 | 3.31 | [M+H]^+^ | 146.0600 | C_9_H_7_NO | -0.21 | 128.0496,118.9676,91.0549 | 2-Hydroxyquinoline^#^ | A | CL |
| 32 | 3.45 | [M+H]^+^ | 138.0913 | C_8_H_11_NO | 0.00 | 121.0651,103.0546,93.0703 | Tyramine^#^ | D | CL |
| 33 | 3.79 | [M+H]⁺ | 268.1039 | C_10_H_13_N_5_O_4_ | -0.90 | 136.0618,119.0355 | Adenosine^#^ | D | CM/CL/RRG |
| 34 | 4.27 | [M+H]^+^ | 252.1089 | C_10_H_13_N_5_O_3_ | -1.10 | 136.0618,117.0550,99.0447 | 2'-Deoxyadenosine^#^ | D | CL |
| 35 | 4.57 | [M+H]^+^ | 284.0987 | C_10_H_13_N_5_O_5_ | -1.00 | 152.0568,135.0301 | Guanosine^#^ | D | CM |
| 36 | 6.10 | [M+H]^+^ | 177.1022 | C_10_H_12_N_2_O | -0.28 | 160.0757,149.0122,131.0016 | 5-Hydroxytryptamine^#^ | D | CL |
| ^√^37 | 7.77 | [M+H]^+^ | 282.1195 | C_11_H_15_N_5_O_4_ | -0.20 | 150.0775,123.0443,72.0815 | 1-Methyladenosine* | D | CM/CL/RRG |
| 38 | 9.74 | [M+H]^+^ | 169.0494 | C_8_H_8_O_4_ | -0.68 | 151.0391,125.0599,107.9602 | Vanillic Acid^#^ | B | CL/RRG |
| 39 | 11.42 | [M+H]^+^ | 251.1388 | C_13_H_18_N_2_O_3_ | -0.83 | 234.1123,163.0389,89.1079,72.0815 | (E)-N-Caffeoylputrescine^#^ | E | CL |
| 40 | 12.10 | [M+H]^+^ | 188.0706 | C_11_H_9_NO_2_ | -0.08 | 170.0590,142.0650,115.0546,91.0547 | 3-Indoleacrylic Acid^#^ | D | CM/CL/RRG |
| 41 | 12.18 | [M+H]^+^ | 355.1019 | C_16_H_18_O_9_ | -1.21 | 163.0390,135.0441,117.0338 | Neochlorogenic Acid^#^ | B | CM/CL |
| 42 | 12.19 | [M-H]^-^ | 353.0884 | C_16_H_18_O_9_ | 4.73 | 191.0556,179.0343,173.0449,135.0440 | Cryptochlorogenic Acid^#^ | B | CM |
| 43 | 12.22 | [M+H]^+^ | 205.0972 | C_11_H_12_N_2_O_2_ | -0.07 | 188.0706,170.0600,159.0917 | L(-)-Tryptophan^#^ | B | CL |
| ^√^44 | 12.30 | [M+H]^+^ | 605.3179 | C_30_H_44_N_4_O_9_ | -0.18 | 313.1395,293.1856,222.25 | N^1^-(3,4,5-Trihydroxycinn-Amoyl)-N^5^-Hydroxyacetyl-N^14^-Dihydrocaffeoylspermine^#^ | E | CL |
| ^√^45 | 13.39 | [M+H]^+^ | 467.1185 | [C_21_H_22_O_12_](https://pubchem.ncbi.nlm.nih.gov/#query=C21H22O12) | 0.15 | 259.0602,153.0182 | Taxifolin-3-Glucoside* | F | CM |
| 46 | 13.47 | [M+H]^+^ | 287.0548 | C_15_H_10_O_6_ | -0.82 | 269.0440,153.0183,123.0443 | Kaempferol^#^ | F | RRG |
| 47 | 13.51 | [M+H]^+^ | 305.0654 | C_15_H_12_O_7_ | -0.49 | 287.0548.259.0608.153.0183.123.0442 | Taxifolin^#^ | F | CM |
| ^√^48 | 13.51 | [M+H]^+^ | 287.0548 | C_15_H_10_O_6_ | 3.86 | 269.0440,241.0495,213.0549,153.0183 | Luteolin^#^ | F | CM/CL |
| 49 | 13.60 | [M-H]^-^ | 339.0727 | C_15_H_16_O_9_ | 4.55 | 177.0186,149.0235,133.0284 | 7-(β-D-Glucopyranosyloxy)-8-Hydroxycoumarin^#^ | G | CM |
| 50 | 13.74 | [M+H]^+^ | 531.3175 | C_28_H_42_N_4_O_6_ | -0.21 | 513.3054,293.1856,222.1124,165.0546,123.0442 | Kukoamine A^#^ | E | CL |
| 51 | 13.91 | [M-H]^-^ | 165.0548 | C_9_H_10_O_3_ | 1.21 | 147.0442,121.0647,93.0333 | Phenyllactic Acid^#^ | B | CM/CL/RRG |
| 52 | 14.07 | [M+H]^+^ | 531.3175 | C_28_H_42_N_4_O_6_ | -0.40 | 513.3035,367.2730,293.1858,222.1125,165.0547,123.0442 | Kukoamine B^▲^ | E | CL |
| ^√^53 | 14.66 | [M+H]^+^ | 325.0915 | C_15_H_16_O_8_ | -0.30 | 163.0390,119.0494,107.0495 | Skimmin^#^ | G | CM |
| 54 | 14.68 | [M+H]^+^ | 265.1545 | C_14_H_20_N_2_O_3_ | -0.19 | 163.0390,145.0285,117.0337,89.0391 | N-Feruloyl Putrescine  ( Subaphylline) ^#^ | E | CL |
| 55 | 14.85 | [M+H]^+^ | 245.0807 | C_14_H_12_O_4_ | -0.55 | 227.0701,161.0597,135.0441,107.0495,86.0970 | Oxyresveratrol^#^ | H | CM |
| 56 | 14.89 | [M+H]^+^ | 341.0865 | C_15_H_16_O_9_ | -0.55 | 179.0339,135.0442,151.0390 | Esculin^#^ | G | CM |
| 57 | 14.97 | [M+H]^+^ | 529.3020 | C_28_H_40_N_4_O_6_ | -0.57 | 293.1857,222.1125,163.0389 | N^1^-Dihydrocaffeoyl-N^10^-  Caffeoylspermine^#^ | E | CL |
| 58 | 14.99 | [M-H]^-^ | 471.1148 | C_20_H_24_O_13_ | 3.87 | 177.0186,149.0233,133.0284 | 7-Hydroxycoumarin-6-O-β-D-Glucopyranosyl-(1→5)-β-D-Apiofuranoside^#^ | G | CM |
| 59 | 15.36 | [M-H]^-^ | 471.1151 | C_20_H_24_O_13_ | 3.68 | 177.0186,149.0235,133.028 | 5-Hydroxycoumarin-7-O-β-D-Glucopyranosyl-(1→5)-β-D-Apiofuranoside^#^ | G | CM |
| 60 | 15.47 | [M-H]^-^ | 485.1311 | C_21_H_26_O_13_ | 4.34 | 177.0186,133.0284,105.0332 | 6-Hydroxycoumarin-7-O-α-L-Rhamnopyranosyl-(1→6)-β-D-Glucopyranoside^#^ | G | CM |
| 61 | 15.54 | [M+H]^+^ | 529.3019 | C_28_H_40_N_4_O_6_ | -0.34 | 293.1858,222.1125,163.0390 | N^1^-Caffeoyl-N^10^-Dihydrocaffeoylspermine^#^ | E | CL |
| ^√^62 | 15.55 | [M-H]^-^ | 455.1203 | C_20_H_24_O_12_ | 4.17 | 161.0235,149.0444,133.0283 | Apiosylskimmin* | G | CM |
| ^√^63 | 15.75 | [M-H]^-^ | 181.0500 | C_9_H_10_O_4_ | 2.35 | 153.915,137.0597,109.0283 | Homovanillic Acid* | B | CM/CL/RRG |
| 64 | 16.12 | [M-H]^-^ | 353.0883 | C_16_H_18_O_9_ | 4.56 | 191.0555,173.0448,161.0235 | Chlorogenic Acid^▲^ | B | CM/CL |
| 65 | 16.15 | [M+H]^+^ | 355.1021 | C_16_H_18_O_9_ | -0.78 | 193.0498,163.0390 | Isoscopoletin^#^ | G | CM/CL |
| 66 | 16.19 | [M-H]^-^ | 471.1150 | C_20_H_24_O_13_ | 3.30 | 177.0185,133.0283 | 6-Hydroxycoumarin-7-O-β-D-Glucopyranosyl-(1→5)-β-D-Apiofuranoside^#^ | G | CM |
| 67 | 16.29 | [M-H]^-^ | 485.1309 | C_21_H_26_O_13_ | 3.96 | 177.0186,133.0284,89.0382 | 5-Hydroxycoumarin-7-O-Α-L-Rhamnopyranosyl-(1→6)-β-D-Glucopyranoside^#^ | G | CM |
| 68 | 16.37 | [M+H]^+^ | 529.3019 | C_28_H_40_N_4_O_6_ | -0.34 | 367.2717,222.1125 | N^1^-Caffeoyl-N^14^-Dihydrocaffeoylspermine^#^ | E | CL |
| 69 | 16.39 | [M+H]^+^ | 355.1020 | C_16_H_18_O_9_ | -0.94 | 193.0497,178.0260,165.0543 | Scopolin^#^ | G | CM/CL |
| 70 | 16.39 | [M-H]^-^ | 567.1725 | C_26_H_32_O_14_ | 3.03 | 405.1197,243.0663,225.0556,215.0707,199.0760 | Mulberroside A^▲^ | H | CM |
| 71 | 16.41 | [M+H]^+^ | 193.0494 | C_10_H_8_O_4_ | -0.65 | 178.0261,165.0548,122.0364 | Daphnetin 7-Methyl Ether^#^ | G | CL |
| 72 | 16.66 | [M-H]^-^ | 153.0184 | C_7_H_6_O_4_ | 1.08 | 109.0283,91.0288 | β-Dihydroxybenzoic Acid^#^ | B | CM/RRG |
| 73 | 17.01 | [M-H]^-^ | 177.0186 | C_9_H_6_O_4_ | 1.89 | 149.0233,133.0283,105.0333,89.0383 | Esculetin^▲^ | G | CM/RRG |
| ^√^74 | 17.33 | [M-H]^-^ | 353.0884 | C_16_H_18_O_9_ | 4.48 | 191.0555,173.0447,135.0440 | Cryptochlorogenic Acid^#^ | B | CM/CL |
| 75 | 17.40 | [M+H]^+^ | 163.0389 | C_9_H_6_O_3_ | -0.37 | 145.0285,135.0441,117.0338 | Umbelliferone^#^ | G | CM/RRG |
| 76 | 17.54 | [M+H]^+^ | 545.3331 | C_29_H_44_N_4_O_6_ | -0.43 | 222.1124,179.0704,123.0442 | N-Dihydroferuloyl-N'-Dihydrocaffeoylspermine^#^ | E | CL |
| ^√^77 | 17.64 | [M+H]^+^ | 229.0859 | C_14_H_12_O_3_ | -0.05 | 211.0753,135.0441 | Resveratrol^#^ | H | CM |
| ^√^78 | 17.76 | [M+H]^+^ | 545.3333 | C_29_H_44_N_4_O_6_ | -0.10 | 222.1125,179.0701,123.0442 | N^1^-Dihydroferuloyl-N^5^-Dihydrocaffeoylspermine^#^ | E | CL |
| 79 | 17.80 | [M-H]^-^ | 179.0343 | C_9_H_8_O_4_ | 2.09 | 135.0440,107.0492 | Caffeic Acid^▲^ | B | CM |
| 80 | 18.27 | [M-H]^-^ | 625.1422 | C_27_H_30_O_17_ | 3.56 | 463.0888,257.0463 | Herbacetin-3,8-Diglucopyranoside^#^ | F | CM |
| ^√^81 | 18.29 | [M+H]^+^ | 465.1028 | C_21_H_20_O_12_ | 0.03 | 303.0499,153.0183,137.0234 | Quercetin-4'-O-Glucoside^*^ | F | CM |
| ^√^82 | 18.37 | [M-H]^-^ | 353.0884 | C_16_H_18_O_9_ | 4.65 | 191.0555,179.0343,173.0448,135.0441 | Caffeoylquinic Acid Isomers^#^ | B | CM |
| 83 | 20.03 | [M+H]^+^ | 474.2597 | C_25_H_35_N_3_O_6_ | -0.26 | 310.2131,236.1277,222.1125,165.0546 | N¹,N^10^-Bis(Dihydrocaffeoyl)Sperm-idine^#^ | E | CL |
| 84 | 20.42 | [M+H]^+^ | 508.3115 | C_24_H_45_NO_10_ | -1.84 | 346.2587,328.2480 | Morusmic acid E^*^ | B | CM |
| ^√^85 | 20.86 | [M+NH_4_]^+^ | 434.2018 | C_19_H_28_O_10_ | -0.67 | 129.0547,85.0290,71.0499 | Hydrangeifolin I^*^ | G | CM |
| 86 | 20.97 | [M-H]^-^ | 415.1043 | C_21_H_20_O_9_ | 4.56 | 295.0616,267.0667,253.0512 | Puerarin^#^ | F | CM/CL |
| 87 | 21.03 | [M-H]^-^ | 565.1572 | C_26_H_30_O_14_ | 3.62 | 403.1039,241.0506 | Mulberroside F^#^ | F | CM |
| ^√^88 | 22.17 | [M+H-H_2_O]^+^ | 135.1168 | C_10_H_16_O | 0.10 | 107.0859,93.0704,79.0548 | (-)-Carveol^*^ | I | CM/CL/RRG |
| 89 | 22.33 | [M+H]^+^ | 472.2441 | C_25_H_33_N_3_O_6_ | -0.28 | 310.2122,220.0968,163.0389 | N¹-Dihydrocaffeoyl-N^5^-Caffeoylspermidine^#^ | E | CL |
| 90 | 23.24 | [M-H]^-^ | 593.1528 | C_27_H_30_O_15_ | 2.73 | 473.1104,383.0779,353.0674,297.0772 | Vicenin II^#^ | F | RRG |
| ^√^91 | 23.29 | [M+H-H_2_O]^+^ | 207.0651 | C_11_H_12_O_5_ | -0.22 | 207.0649,175.0390,147.0441,119.0494 | 4-Hydroxy-3,5-Dimethoxycinnamic Acid* | B | RRG |
| 92 | 23.36 | [M-H]^-^ | 405.1197 | C_20_H_22_O_9_ | 4.08 | 243.0663,225.0555,199.0759,175.0756 | Oxyresveratrol-3-O-β-Glucoside or Its Isomers^#^ | H | CM/RRG |
| 93 | 23.47 | [M+H]^+^ | 472.2442 | C_25_H_33_N_3_O_6_ | 0.04 | 310.2119,222.1125,163.0390 | N^1^-Caffeoyl-N^10^-dihydrocaffeoylspermidine^#^ | E | CL |
| 94 | 23.63 | [M+H]^+^ | 573.3281 | C_30_H_44_N_4_O_7_ | -0.26 | 409.2856,335.1961,293.1856,222.1124 | N^1^-(3-Acetyldihydrocaffeoyl)-N^10^-Dihydrocaffeoylspermine^#^ | E | CL |
| 95 | 23.76 | [M+H]^+^ | 451.1234 | C_21_H_22_O_11_ | -0.24 | 289.0705,271.0599,153.0183 | Maritine^#^ | F | CM |
| ^√^96 | 23.76 | [M+H]^+^ | 289.0704 | C_15_H_12_O_6_ | -0.24 | 153.0184,97.0288 | 2',5,6',7-Tetrahydroxyflavanone^*^ | F | RRG |
| 97 | 24.20 | [M-H]^-^ | 163.0392 | C_9_H_8_O_3_ | 1.41 | 119.049,93.0332 | Cis-4-Coumaric Acid^▲^ | B | CL/RRG |
| ^√^98 | 24.29 | [M+H]^+^ | 479.1183 | C_22_H_22_O_12_ | -0.24 | 317.0655,285.0391,257.0439,153.0182 | Isorhamnetin 3-Galactoside^*^ | F | CL |
| ^√^99 | 24.40 | [M-H]^-^ | 625.1420 | C_27_H_30_O_17_ | 3.27 | 463.0890,301.0357,151.0027 | Quercetin 3,4'-Diglucoside^*^ | F | CM |
| 100 | 24.59 | [M+H]^+^ | 451.1232 | C_21_H_22_O_11_ | -0.64 | 367.7489,289.0705,153.0183 | Eriodictyol-7-O-Glucoside^*^ | F | CM |
| 101 | 24.59 | [M-H]^-^ | 433.1148 | C_21_H_22_O_10_ | 4.29 | 271.0616,151.0027,119.0490,83.0122 | Prunin^#^ | F | RRG |
| 102 | 24.98 | [M-H]^-^ | 405.1198 | C_20_H_22_O_9_ | 4.37 | 243.0663,225.0555,199.0760 | Oxyresveratrol-3-O-β-Glucoside or Its Isomers^#^ | H | CM/RRG |
| 103 | 25.98 | [M-H]^-^ | 463.0890 | C_21_H_20_O_12_ | 4.08 | 301.0357,151.0027 | Isohyperoside^▲^ | F | CM |
| ^√^104 | 26.28 | [M-H]^-^ | 563.1412 | C_26_H_28_O_14_ | 2.19 | 545.1317,473.1093,443.0989,413.0888,383.0777,353.0671,325.0719,297.0770 | Vicenin III^*^ | F | RRG |
| 105 | 26.29 | [M-H]^-^ | 563.1414 | C_26_H_28_O_14_ | 3.35 | 473.1106,443.0995,383.0776,353.0671 | Schaftoside^#^ | F | RRG |
| 106 | 26.47 | [M-H]^-^ | 191.0344 | C_10_H_8_O_4_ | 2.59 | 176.0107,148.0156,104.0254 | Scopoletin^▲^ | G | CM |
| 107 | 27.04 | [M-H]^-^ | 193.0501 | C_10_H_10_O_4_ | 2.98 | 178.0265,149.0598,134.03619 | Ferulic Acid^#^ | B | CL/RRG |
| ^√^108 | 27.23 | [M-H]^-^ | 563.1417 | C_26_H_28_O_14_ | 3.78 | 473.1089,443.0994,383.0787,353.0674 | Isoschaftoside^#^ | F | RRG |
| 109 | 27.32 | [M+H]^+^ | 417.1180 | C_21_H_20_O_9_ | -0.14 | 285.2623,255.0651,185.5358 | Daidzoside^*^ | F | RRG |
| 110 | 27.69 | [M+H]^+^ | 502.2914 | C_27_H_39_N_3_O_6_ | 0.43 | 222.1124 | N^1^, N^5^-Bis ( Dihydroferuloyl) Spermidine^#^ | E | CL |
| 111 | 28.05 | [M-H]^-^ | 549.1624 | C_26_H_30_O_13_ | 3.79 | 429.1057,255.0664,153.0183,135.0077,119.0490,91.0176 | Neoliquiritinapioside^#^ | F | RRG |
| 112 | 28.07 | [M+H]^+^ | 447.1283 | C_22_H_22_O_10_ | -0.27 | 285.0756,270.0522,253.0494 | Calycosin 7-O-Glucoside^#^ | F | RRG |
| 113 | 28.27 | [M+H]^+^ | 302.1385 | C_17_H_19_NO_4_ | -0.55 | 165.0552,138.0913,121.0650,93.0704 | N-Dihydrocaffeoyltyramine^#^ | E | CL |
| 114 | 28.31 | [M+H]^+^ | 695.3648 | C_37_H_50_N_4_O_9_ | -0.35 | 511.4874,293.1857,222.1125 | Tris(Dihydrocaffeoyl)Spermine^#^ | E | CL |
| 115 | 28.47 | [M+H]^+^ | 419.1335 | C_21_H_22_O_9_ | -0.21 | 257.0808,147.0441,137.0234,119.0494 | Neoliquiritin^#^ | F | CM/RRG |
| ^√^116 | 28.53 | [M+H]^+^ | 257.0806 | C_15_H_12_O_4_ | -0.92 | 239.0698,197.0600,183.0803.155.0335,147.0441,137.0234 | Pinocembrin^#^ | F | RRG |
| 117 | 28.54 | [M-H]^-^ | 431.0991 | C_21_H_20_O_10_ | 4.15 | 341.0670,311.0565,269.0462,135.0072,117.0336 | Vitexin^#^ | F | CL |
| 118 | 28.56 | [M-H]^-^ | 549.1624 | C_26_H_30_O_13_ | 3.90 | 531.1040,417.1233,297.0779,255.0665,135.0077,119.0490 | Liquiritin apioside^#^ | F | RRG |
| 119 | 28.75 | [M-H]^-^ | 577.1572 | C_27_H_30_O_14_ | 3.45 | 487.1266,457.1151,383.0778,353.0675 | Violanthin^#^ | F | RRG |
| 120 | 28.84 | [M-H]^-^ | 609.1475 | C_27_H_30_O_16_ | 4.12 | 300.0277,243.0657 | Rutin^▲^ | F | CM/CL/RRG |
| 121 | 28.89 | [M-H]^-^ | 431.0993 | C_21_H_20_O_10_ | 4.77 | 311.0568,269.0458,89.0231 | Isovitexin^*^ | F | CL/RRG |
| ^√^122 | 29.34 | [M+H]^+^ | 273.0754 | C_15_H_12_O_5_ | -0.34 | 163.0390,137.0233,117.0335 | 2',3,4,4'-Tetrahydroxy Chalcone^*^ | F | RRG |
| ^√^123 | 30.20 | [M-H]^-^ | 449.1097 | C_21_H_22_O_11_ | 4.30 | 241.0507,204.9212,199.0395,155.1063 | Astilbin^#^ | F | CM |
| ^√^124 | 31.35 | [M-H]^-^ | 515.1201 | C_25_H_24_O_12_ | 3.22 | 353.0883,191.0555,173.0447 | Isochlorogenic Acid B^#^ | B | CM |
| ^√^125 | 31.62 | [M-H]^-^ | 579.2098 | C_28_H_36_O_13_ | 4.53 | 417.1562,181.0498,137.0236 | Acanthoside B^*^ | D | CM/CL/RRG |
| ^√^126 | 31.95 | [M-H]^-^ | 515.1204 | C_25_H_24_O_12_ | 3.80 | 353.0884,191.0555,179.0341 | Isochlorogenic Acid A^#^ | B | RRG |
| 127 | 32.17 | [M-H]^-^ | 187.0969 | C_9_H_16_O_4_ | 2.43 | 169.0864,125.0960,97.0646 | Azelaic Acid^#^ | B | CM |
| 128 | 32.22 | [M-H]^-^ | 447.0939 | C_21_H_20_O_11_ | 3.76 | 285.0406,151.0027 | Luteoloside^#^ | F | RRG |
| 129 | 32.22 | [M+H]^+^ | 300.1228 | C_17_H_17_NO_4_ | -0.88 | 163.0390,145.0284,135.0441,117.0338 | N-Caffeoyltyramine^#^ | E | CL |
| 130 | 32.36 | [M-H]^-^ | 433.1147 | C_21_H_22_O_10_ | 3.99 | 271.0617,151.0027,119.0490 | Choerospondin^#^ | F | RRG |
| 131 | 32.38 | [M+H]^+^ | 273.0755 | C_15_H_12_O_5_ | -1.03 | 153.0183,147.0440,119.0494 | Naringenin^*^ | F | RRG |
| ^√^132 | 32.64 | [M+H]^+^ | 316.1540 | C_18_H_21_NO_4_ | -1.19 | 280.2626,121.0650 | Dihydro-Feruloyltyramine^#^ | E | CL |
| ^√^133 | 32.85 | [M-H]^-^ | 515.1202 | C_25_H_24_O_12_ | 3.58 | 353.0883,173.0447,135.0440 | Cynarine^*^ | B | CM |
| 134 | 33.18 | [M-H]^-^ | 872.3610 | C_42_H_51_N_9_O_12_ | 4.15 | 842.3536,501.2115,484.1850 | Lyciumin A^#^ | J | CL |
| ^√^135 | 33.16 | [M-H]^-^ | 445.1142 | C_22_H_22_O_10_ | 2.80 | 283.0615,268.0380,224.0477,175.0026 | Sissotrin^*^ | F | RRG |
| 136 | [33.74](https://pubchem.ncbi.nlm.nih.gov/#query=C21H22O9) | [M+H]^+^ | 419.1333 | C_21_H_22_O_9_ | -0.93 | 257.0806,147.0440,137.0233,119.0494 | Isoliquiritin^#^ | F | RRG |
| 137 | 33.74 | [M-H]^-^ | 549.1622 | C_26_H_30_O_13_ | 3.57 | 255.0665,153.0183,119.0490 | Liguiritigenin-7-O-D-Apiosyl-4'-O-D-Glucoside^#^ | F | RRG |
| 138 | 34.12 | [M+H]^+^ | 431.1335 | C_22_H_22_O_9_ | -0.35 | 269.0807,254.0572,237.0546,226.0623,  137.0238 | Ononin^▲^ | F | RRG |
| 139 | 34.21 | [M-H]^-^ | 549.1622 | C_26_H_30_O_13_ | 4.03 | 255.0664,153.0184,135.0076 | Choerospondin Isomers^#^ | F | RRG |
| 140 | 34.36 | [M-H]^-^ | 433.1145 | C_21_H_22_O_10_ | 3.64 | 271.0616,151.0028,119.0490 | Isoliquiritin Apioside^#^ | F | RRG |
| 141 | 34.42 | [M+H]^+^ | 419.1334 | C_21_H_22_O_9_ | -0.30 | 257.0807,239.0701,211.0755,147.0441,137.0234,119.0494 | Liquiritin^▲^ | F | RRG |
| 142 | 34.71 | [M+H]^+^ | 284.1279 | C_17_H_17_NO_3_ | -0.77 | 164.0705,147.0441,121.0650,93.0704 | N-Cumaroyltyramin^#^ | E | CL |
| 143 | 34.83 | [M-H]^-^ | 253.0508 | C_15_H_10_O₄ | 4.92 | 135.0076,117.0333,91.0175 | 7,4'-Dihydroxyflavone^#^ | F | RRG |
| 144 | 34.85 | [M+H]^+^ | 255.0650 | C_15_H_10_O_4_ | -0.77 | 137.0234,119.0493 | Daidzein^#^ | F | RRG |
| 145 | 34.99 | [M+H]^+^ | 419.1334 | C_21_H_22_O_9_ | -0.24 | 257.0807,147.0441,137.0233,123.0443 | Neoisoliquiritin^*^ | F | RRG |
| 146 | 35.09 | [M-H]^-^ | 301.0357 | C_15_H_10_O_7_ | 4.85 | 151.0028,125.0233,107.0123 | Morin^#^ | F | CM |
| 147 | 35.40 | [M+H]^+^ | 314.1385 | C_18_H_19_NO_4_ | -0.72 | 177.0546,149.0602,145.0284,121.0650 | N-Feruloyltyramine^*^ | E | CL |
| 148 | 35.46 | [M+H]^+^ | 461.1441 | C_23_H_24_O_11_ | -0.33 | 299.0913,284.0678 | 4',6-Dimethylisoflavone-7-O-β -Glucoside^#^ | F | RRG |
| 149 | 35.76 | [M-H]^-^ | 255.0664 | C_15_H_12_O_4_ | 4.84 | 153.0184,135.0076,119.0490,91.0175 | Isoliquiritigenin^#^ | F | RRG |
| 150 | 35.78 | [M-H]^-^ | 895.3770 | C_44_H_52_N_10_O_11_ | 4.11 | 865.3713,687.2930,471.1991,281.1254,153.0661 | Lyciumin B^#^ | J | CL |
| 151 | 35.88 | [M-H]^-^ | 201.1127 | C_10_H_18_O_4_ | 2.81 | 183.1019,157.1228,139.1118,111.0803 | Decanedioicacid^#^ | B | CM/RRG |
| 152 | 36.03 | [M-H]^-^ | 299.0564 | C_16_H_12_O_6_ | 4.53 | 284.0330,256.0378 | Chrysoeriol^#^ | F | RRG |
| ^√^153 | 36.15 | [M-H]^-^ | 283.0615 | C₁₆H₁₂O₅ | 4.91 | 268.038,239.0349,240.0425,211.0396 | Acacetin^#^ | F | RRG |
| 154 | 36.26 | [M-H]^-^ | 255.0664 | C_15_H_12_O_4_ | 1.21 | 153.0184,135.0076,119.0490,91.0176 | Liquiritigenin^#^ | F | RRG |
| 155 | 36.34 | [M-H]^-^ | 962.4075 | C_49_H_57_N_9_O_12_ | 3.18 | 932.3939,754.3217,724.3164,591.2485,561.2472,506.2427,387.1683 | Lyciumin D^#^ | J | CL |
| ^√^156 | 36.46 | [M+H]^+^ | 461.1441 | C_23_H_24_O_11_ | -0.15 | 299.0913,284.0679 | 4',6-Dimethylisoflavone-7-O-β-Glucoside Isomer ^#^ | F | RRG |
| 157 | 36.49 | [M-H]^-^ | 983.4519 | C_48_H_72_O_21_ | 3.73 | 821.3923,645.3710,351.0574,193.0347 | Licoricesaponin A3^#^ | I | RRG |
| ^√^158 | 36.94 | [M-H]^-^ | 879.4043 | C_44_H_64_O_18_ | 3.88 | 351.0578,193.0351,113.0232 | 22β-Acetoxyl-Glycyrrhizin^#^ | I | RRG |
| 159 | 37.02 | [M+H]^+^ | 487.3413 | C_30_H_46_O_5_ | -0.95 | 187.1482,119.0858 | Quillaic Acid^#^ | I | RRG |
| 160 | 37.03 | [M-H]^-^ | 837.3937 | C_42_H_62_O_17_ | 4.01 | 351.0560,193.0355,113.0232 | Licoricesaponin G2^#^ | I | RRG |
| 161 | 37.17 | [M-H]^-^ | 227.1287 | C_12_H_20_O_4_ | 3.98 | 209.1179,183.0384 | Traumatic Acid^*^ | B | CM/CL |
| 162 | 37.34 | [M-H]^-^ | 215.1286 | C_11_H_20_O_4_ | 3.88 | 197.1178,153.1276 | Undecanedioic Acid^#^ | B | CM/CL/RRG |
| 163 | 37.37 | [M+H]^+^ | 271.0962 | C_16_H_14_O_4_ | -0.98 | 253.0847,134.0363,121.0286 | Retrochalcone^#^ | F | RRG |
| 164 | 37.47 | [M+H]^+^ | 271.0598 | C_15_H_10_O_5_ | -1.07 | 253.0487,243.0627,229.0862,225.0544 | Apigenin^#^ | F | CL |
| 165 | 37.51 | [M+H]^+^ | 839.4055 | C_42_H_62_O_17_ | -0.52 | 663.3754,645.3614,487.3416,469.3312,451.3206,439.3217 | Licoricesaponin G2 Isomer^#^ | I | RRG |
| 166 | 37.61 | [M-H]^-^ | 819.3833 | C_42_H_60_O_16_ | 4.32 | 351.0561,193.0346,175.0239,113.0232 | Licoricesaponin E2^#^ | I | RRG |
| 167 | 37.87 | [M+H]^+^ | 823.4106 | C_42_H_62_O_16_ | -0.51 | 647.3794,471.3463,453.3363,435.3250,407.3294 | Glycyrrhizic Acid^▲^ | I | RRG |
| 168 | 37.96 | [M-H]^-^ | 807.4191 | C_42_H_64_O_15_ | 3.62 | 351.0586,193.353,113.0231,71.0124 | Licoricesaponin B2^#^ | I | RRG |
| ^√^169 | 38.02 | [M-H]^-^ | 339.1240 | C₂₀H₂₀O₅ | 3.89 | 177.0913,161.0235,135.0440 | Euchrenone A^#^ | F | RRG |
| ^√^170 | 38.07 | [M+H]^+^ | 423.3620 | C_30_H_46_O | -0.41 | 229.1958,203.1794,133.1012,95.0861 | Glochidone^*^ | I | CM/ RRG |
| ^√^171 | 38.25 | [M-H]^-^ | 821.3990 | C_42_H_62_O_16_ | 4.36 | 498.7736,351.0581,193.0343,113.0232 | Uralsaponina A^#^ | I | RRG |
| 172 | 38.26 | [M+H]^+^ | 471.3466 | C_30_H_46_O_4_ | -0.56 | 453.3375,435.3228 | 18β-Glycyrrhetinic Acid^#^ | I | RRG |
| 173 | 38.26 | [M+H]^+^ | 823.4104 | C_42_H_62_O_16_ | -0.80 | 647.3739,471.3460 | (18β,20α)-Glycyrrhizic Acid^#^ | I | RRG |
| 174 | 38.37 | [M+H]^+^ | 823.4105 | C_42_H_62_O_16_ | -0.66 | 647.3739,471.3460 | Licoricesaponin K2^#^ | I | RRG |
| 175 | 38.42 | [M+H]^+^ | 269.0806 | C_16_H_12_O_4_ | -0.97 | 254.0577,237.0547,226.0646 | Formononetin^▲^ | F | RRG |
| ^√^176 | 38.48 | [M+H]^+^ | 274.2738 | C_16_H_35_NO_2_ | -0.23 | 106.0866,88.0763,70.0659 | N-Lauryldiethanolamine^*^ | D | CM/CL/RRG |
| 177 | 38.50 | [M-H]^-^ | 271.0978 | C_16_H_16_O_4_ | 1.36 | 135.0439 | Vestitol^#^ | F | RRG |
| 178 | 38.52 | [M-H]^-^ | 823.4143 | C_42_H_64_O_16_ | 3.94 | 471.3497,351.0576,193.0348,113.0232 | Licoricesaponin J2^#^ | I | RRG |
| 179 | 38.68 | [M-H]^-^ | 367.1191 | C_21_H_20_O_6_ | 3.99 | 309.0412,283.0247,135.0077 | Glicoricone^#^ | I | RRG |
| ^√^180 | 38.84 | [M+H-H_2_O]^+^ | 439.3565 | C_30_H_48_O_3_ | -1.18 | 421.1646,403.1539,189.1636,153.0182 | 3-Hydroxyolean-12-En-29-Oic Acid^*^ | I | RRG |
| 181 | 38.86 | [M-H]^-^ | 807.4191 | C_42_H_64_O_15_ | 3.69 | 631.3916,351.0570,193.0348,113.0231 | Licoricesaponin B2 Isomer^#^ | I | RRG |
| 182 | 38.92 | [M+H]^+^ | 627.1859 | C_35_H_30_O_11_ | -0.18 | 499.1390,433.0916,153.0183,137.0233 | Kuwanon L^#^ | F | CL |
| 183 | 39.09 | [M-H]^-^ | 707.2153 | C_40_H_36_O_12_ | 4.28 | 597.1785,353.1042,135.0441 | Sanggenon C^#^ | F | CM |
| 184 | 39.18 | [M+H]^+^ | 271.0960 | C_16_H_14_O_4_ | -0.45 | 271.0963,161.0598,137.0597 | Medicarpin^#^ | F | RRG |
| ^√^185 | 39.19 | [M-H]^-^ | 353.1037 | C_20_H_18_O_6_ | 4.91 | 231.0662,151.0028,125.0233 | Gancaonin C^#^ | F | RRG |
| 186 | 39.33 | [M-H]^-^ | 353.1036 | C_20_H_18_O_6_ | 4.66 | 227.0711,201.0372,125.0233, | Sangenon F or its isomers^#^ | F | RRG |
| 187 | 39.40 | [M-H]^-^ | 367.1192 | C_21_H_20_O_6_ | 4.32 | 309.0410,297.0411,201.0183,148.0157 | Glycycoumarin^#^ | G | RRG |
| ^√^188 | 39.44 | [M-H]^-^ | 437.1610 | C_25_H_26_O_7_ | 3.57 | 368.1225,151.0025,125.0232 | Sanggenon N^#^ | F | CM |
| ^√^189 | 39.52 | [M+H]^+^ | 357.1692 | C_20_H_20_O_6_ | -1.21 | 301.1068.153.0547 | Sigmoidin B^#^ | F | RRG |
| ^√^190 | 39.63 | [M-H]^-^ | 353.1035 | C_20_H_18_O_6_ | 4.41 | 151.0756,125.0232 | Licoisoflavanone^#^ | F | RRG |
| 191 | 39.65 | [M-H]^-^ | 691.2201 | C_40_H_36_O_11_ | 3.91 | 581.1830,419.1501,379.1196,353.1051,151.0388,109.0282 | Kuwanon G^#^ | F | CM |
| ^√^192 | 39.71 | [M-H]^-^ | 351.0880 | C_20_H_16_O_6_ | 4.66 | 199.0760,151.0027 | Licoisoflavone B^#^ | F | RRG |
| ^√^193 | 39.71 | [M-H]^-^ | 645.3658 | C_36_H_54_O_10_ | 3.81 | 469.3346, | Glycyrrhetic Acid 3-O-Glucuronide^#^ | I | RRG |
| 194 | 39.75 | [M-H]^-^ | 353.1037 | C_20_H_18_O_6_ | 5.00 | 297.0407,227.0711,125.0232 | Licoflavonol^#^ | F | RRG |
| ^√^195 | 39.77 | [M-H]^-^ | 353.1036 | C₂₀H₁₈O₆ | 4.58 | 227.0711,201.0923,125.0232 | Sangenon F or its isomers^#^ | F | CM |
| ^√^196 | 39.98 | [M-H]^-^ | 353.1036 | C_20_H_18_O_6_ | 4.66 | 297.0407,227.0354,125.0232 | Licoisoflavone A^#^ | F | RRG |
| ^√^197 | 40.08 | [M-H]^-^ | 353.1036 | C_20_H_18_O_6_ | 4.66 | 227.0712,125.0232 | 5'-(3-Methyl-2-Butenyl)-3',4',5,7-Tetrahydroxyisoflavone^#^ | F | RRG |
| ^√^198 | 40.10 | [M-H]^-^ | 381.1349 | C_22_H_22_O₆ | 4.26 | 351.0881,297.0408 | Licoricone^#^ | F | RRG |
| ^√^199 | 40.28 | [M+H]⁺ | 288.2531 | C_16_H_33_NO_3_ | -0.94 | 227.2005,106.0866,88.0762,70.0659 | N, N-Bis(2-Hydroxyethyl) Dodecanamide^*^ | E | CM/CL/RRG |
| ^√^200 | 40.18 | [M-H]^-^ | 437.1612 | C_25_H_26_O_7_ | 3.91 | 257.0822,191.0707,125.0232 | Morusinol, Mulberranol, Kuwanon U^#^ | F | CM |
| ^√^201 | 40.30 | [M+H]^+^ | 235.1691 | C_15_H_22_O_2_ | -0.54 | 189.1637,161.0959,133.1012119.0857 | Curcumenol^*^ | I | CM/CL/RRG |
| ^√^202 | 40.37 | [M+H]^+^ | 423.1798 | C_25_H_26_O_6_ | -0.38 | 367.1179,311.0549,283.0599,241.0494 | Mulberrin C^#^ | F | CM |
| 203 | 40.93 | [M-H]^-^ | 351.0880 | C_20_H_16_O_6_ | 4.66 | 283.0981,275.5970,199.0763 | Semilicoisoflavone B^#^ | F | RRG |
| ^√^204 | 41.26 | [M-H]^-^ | 335.0930 | C_20_H_16_O_5_ | 4.75 | 305.0467,291.0663,151.0026 | Glabrone^#^ | F | RRG |
| ^√^205 | 41.28 | [M-H]^-^ | 419.1508 | C_25_H_24_O_6_ | 4.40 | 231.0661,151.0027,125.0234 | Sanggenon V^#^ | F | CM |
| ^√^206 | 41.33 | [M-H]^-^ | 419.1507 | C_25_H_24_O_6_ | 4.26 | 231.0661,151.0027,125.0234 | Kuwanon A^#^ | F | CM |
| ^√^207 | 41.75 | [M+H]^+^ | 391.2838 | C_24_H_38_O_4_ | -0.51 | 167.0339,149.0234,121.0285 | Bis(2-Ethylhexyl) Phthalate^*^ | D | CM/CL/RRG |
| ^√^208 | 41.75 | [M+H]^+^ | 315.2525 | C_18_H_34_O_4_ | -0.49 | 259.1904,203.1278,139.1118,121.1015 | Dibutyl Decanedioate^*^ | D | CM/CL/RRG |
| 209 | 41.75 | [M+H]^+^ | 279.2316 | C_18_H_30_O_2_ | -0.23 | 243.2115,109.1015,95.0860,81.0705 | α-Linolenic Acid^*^ | D | CM |
| ^√^210 | 41.92 | [M-H]^-^ | 421.1664 | C_25_H_26_O_6_ | 4.31 | 269.1555,151.0026,125.0232 | Kuwanon T^#^ | F | CM |
| 211 | 42.10 | [M-H]^-^ | 419.1506 | C_25_H_24_O_6_ | 4.09 | 297.1135,217.0501,201.0925,191.0706,109.0283 | Morusin^#^ | F | CM |
| 212 | 42.46 | [M-H]^-^ | 391.1921 | C_25_H_28_O_4_ | 4.48 | 307.0982,254.1318 | Mulberrofuran N, A, B^#^ | F | CM |
| ^√^213 | 42.68 | [M+H]^+^ | 371.3153 | C_22_H_42_O_4_ | -0.28 | 147.0652,129.0547,101.0601 | Bis(2-Ethylhexyl) Adipate^*^ | D | CM/CL/RRG |
| 214 | 42.76 | [M+H]^+^ | 279.1589 | C_16_H_22_O_4_ | -0.63 | 205.0859,149.0234,121.0287,57.0707 | Diisobutyl Phthalate^#^ | D | CM |
| ^√^215 | 43.67 | [M+H]^+^ | 403.2322 | C_20_H_34_O_8_ | -1.08 | 217.0346,157.0132,129.0183 | Tributyl Acetylcitrate^*^ | D | CM/CL/RRG |
| ^√^216 | 44.08 | [M+H]^+^ | 363.3099 | C_20_H_42_O_5_ | -0.56 | 195.1227,133.0860,89.0603 | Dodecyltetraglycol^*^ | D | CM/CL/RRG |
| ^√^217 | 44.83 | [M+H-H_2_O]^+^ | 439.3566 | C_30_H_48_O_3_ | -0.46 | 393.3505,259.1699,137.1325,95.0860 | 3-Epi-Betulinic Acid^*^ | I | CM |
| 218 | 45.18 | [M+H]^+^ | 256.2632 | C_16_H_33_NO | -1.10 | 211.5999,102.0918,130.1227,88.0762 | Hexadecanamide^#^ | E | CL |

Note: *t*_R_: retention time; A: Alkaloids; B: Organic acids; C: Saccharides; D: Other categories; E: Amides; F: Flavonoids; G: Coumarins; H: Stilbenes; I: Terpenoids; J: Cyclic peptides. ^√^：First reported in XBSD; ^▲^: level 1; ^#^: level 2; *:level 3; CM: Cortex Mori; CL: Cortex Lycii; RRT:Radix et Rhizoma Glycyrrhizae.

# Supplementary Figures


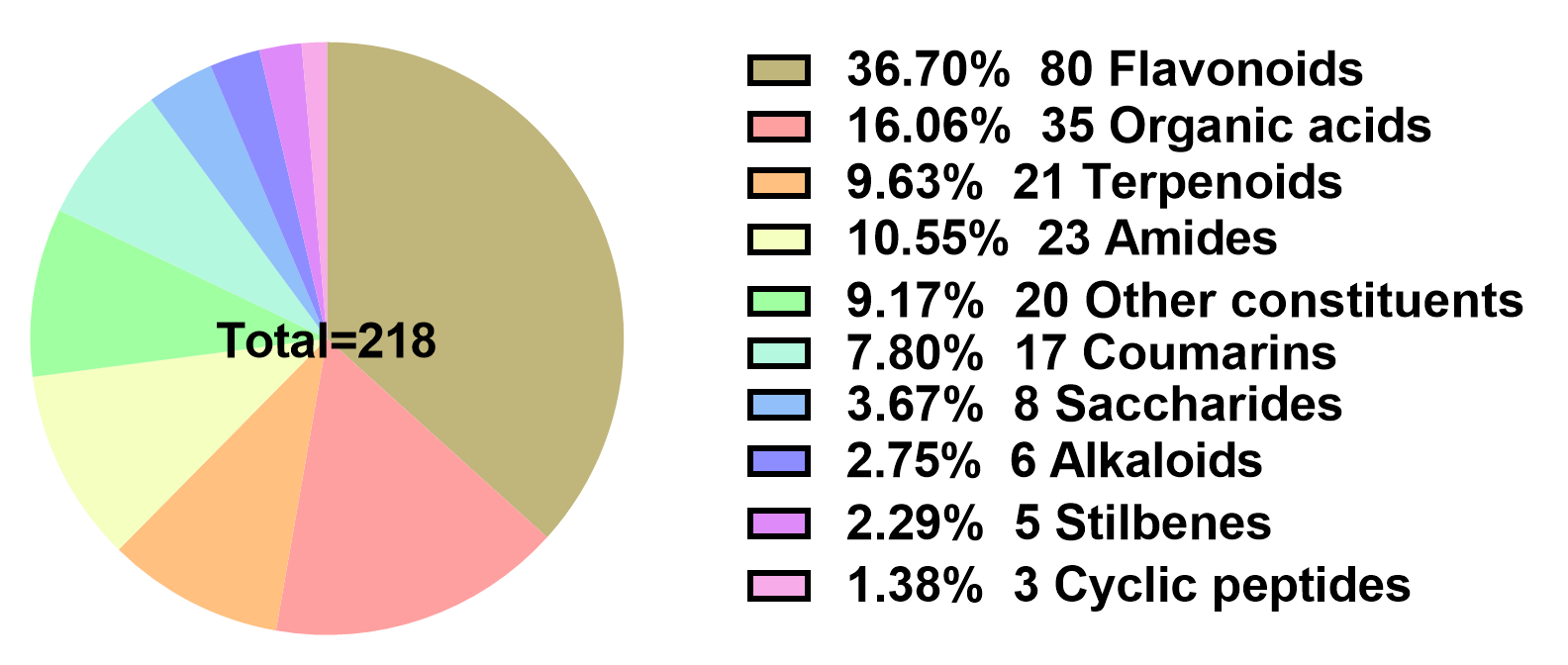


**Supplementary Figure S1.** Structural classification of the annotated compounds from XBSD.

**
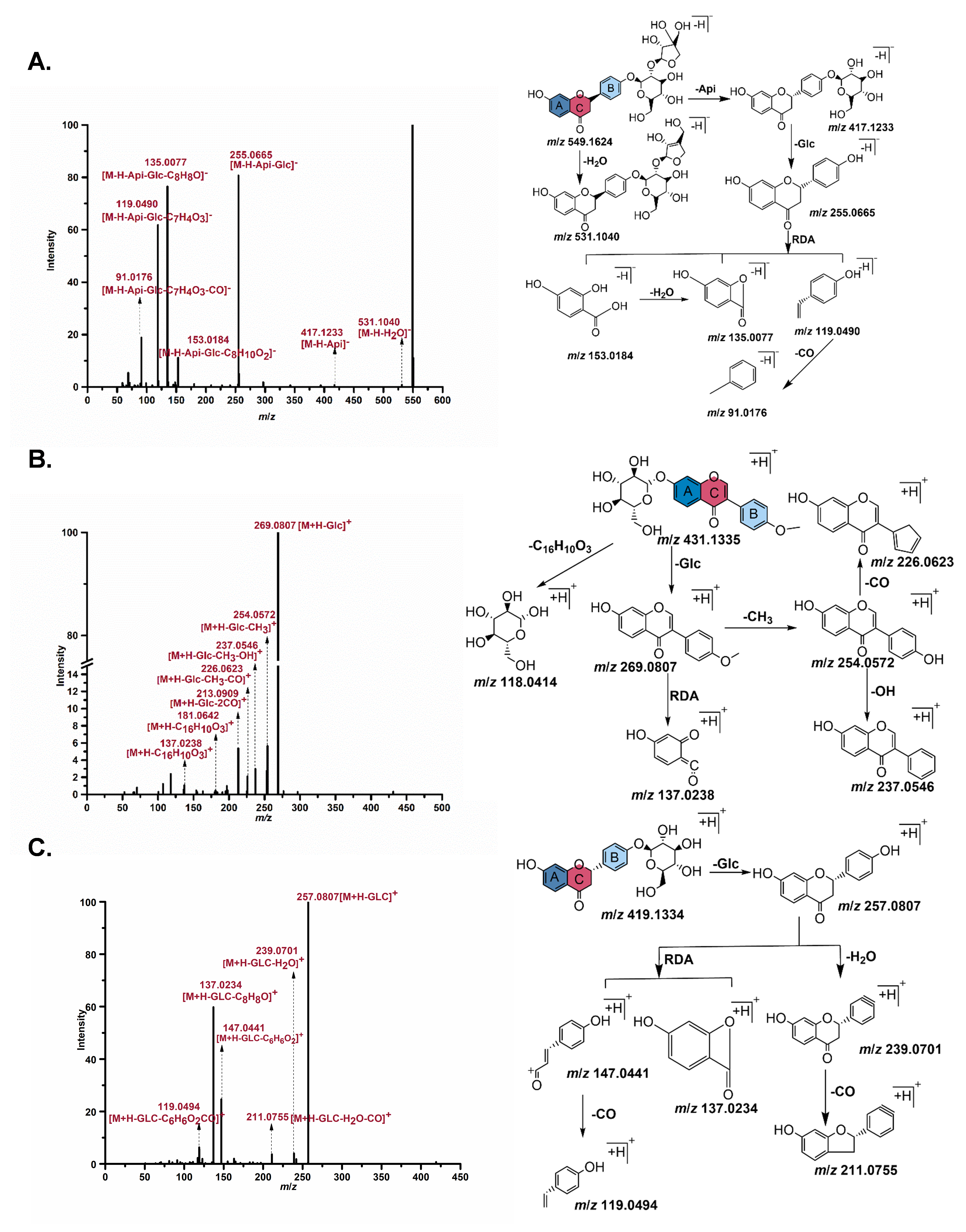
**

**Supplementary Figure S2.** MS/MS spectra and proposed fragmentation pathways of liquiritin apioside (A), ononin (B), and liquiritin (C) from XBSD.


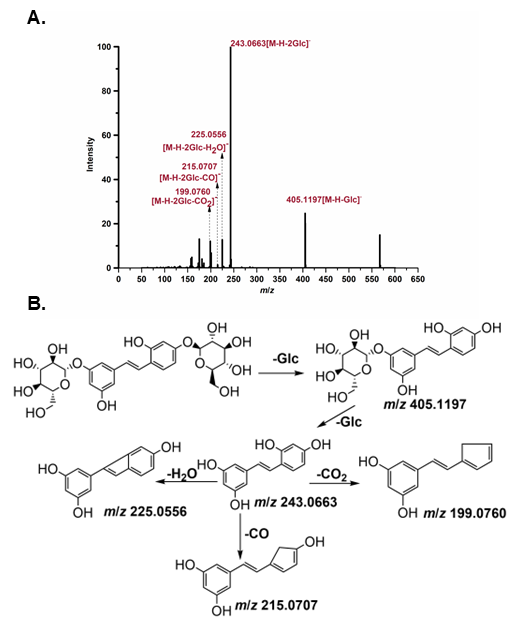


**Supplementary Figure S3.** MS/MS spectrum and proposed fragmentation pathways of mulberroside A from XBSD.


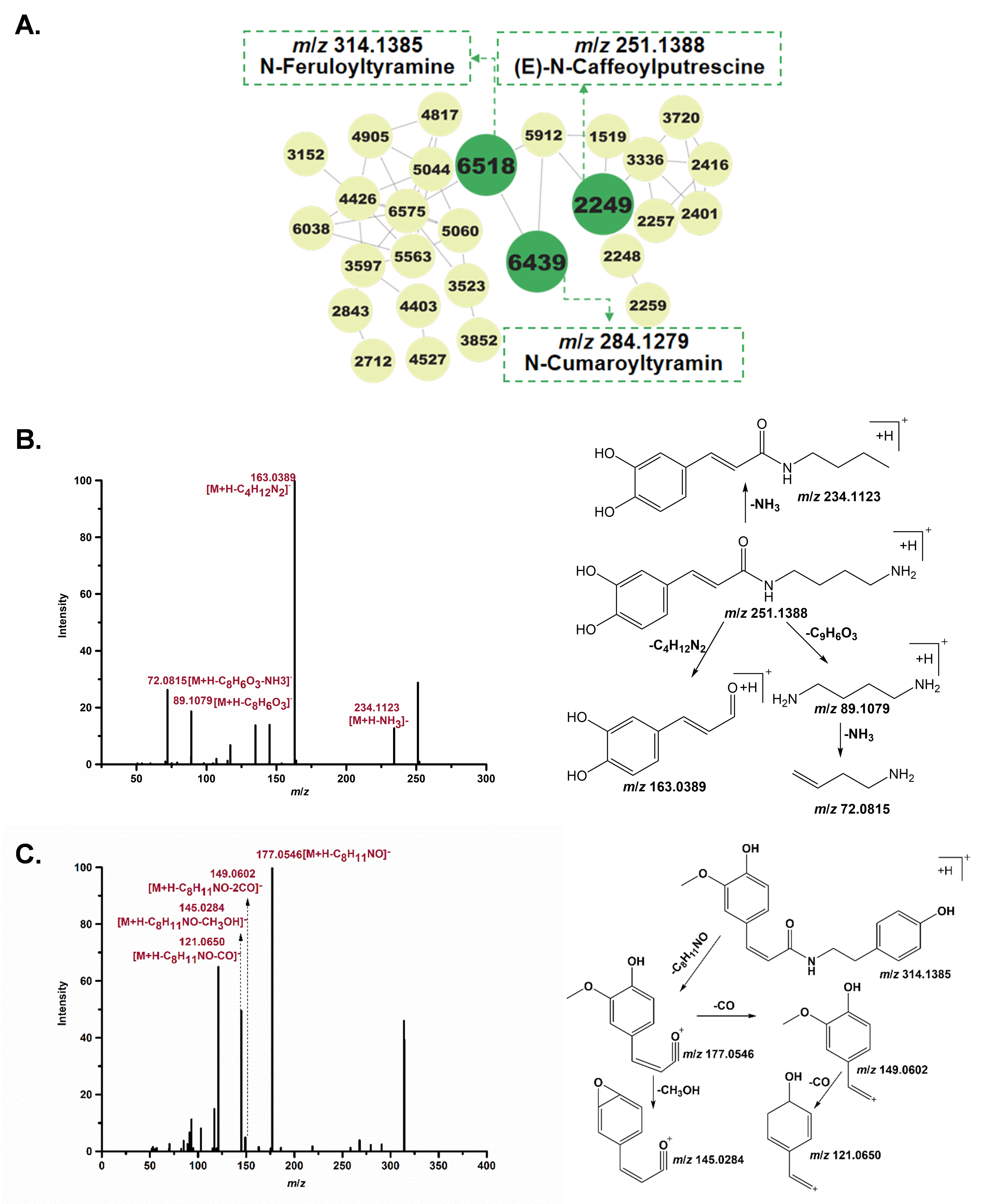


**Supplementary Figure S4.** Feature-based molecular networking and proposed fragmentation pathways of amide compounds from XBSD. (A) Molecular network analysis of N-coumaroyltyramine, (E)-N-caffeoylputrescine, and N-feruloyltyramine. (B) MS/MS spectrum and proposed fragmentation pathways of N-coumaroyltyramine. (C) MS/MS spectrum and proposed fragmentation pathways of N-feruloyltyramine.


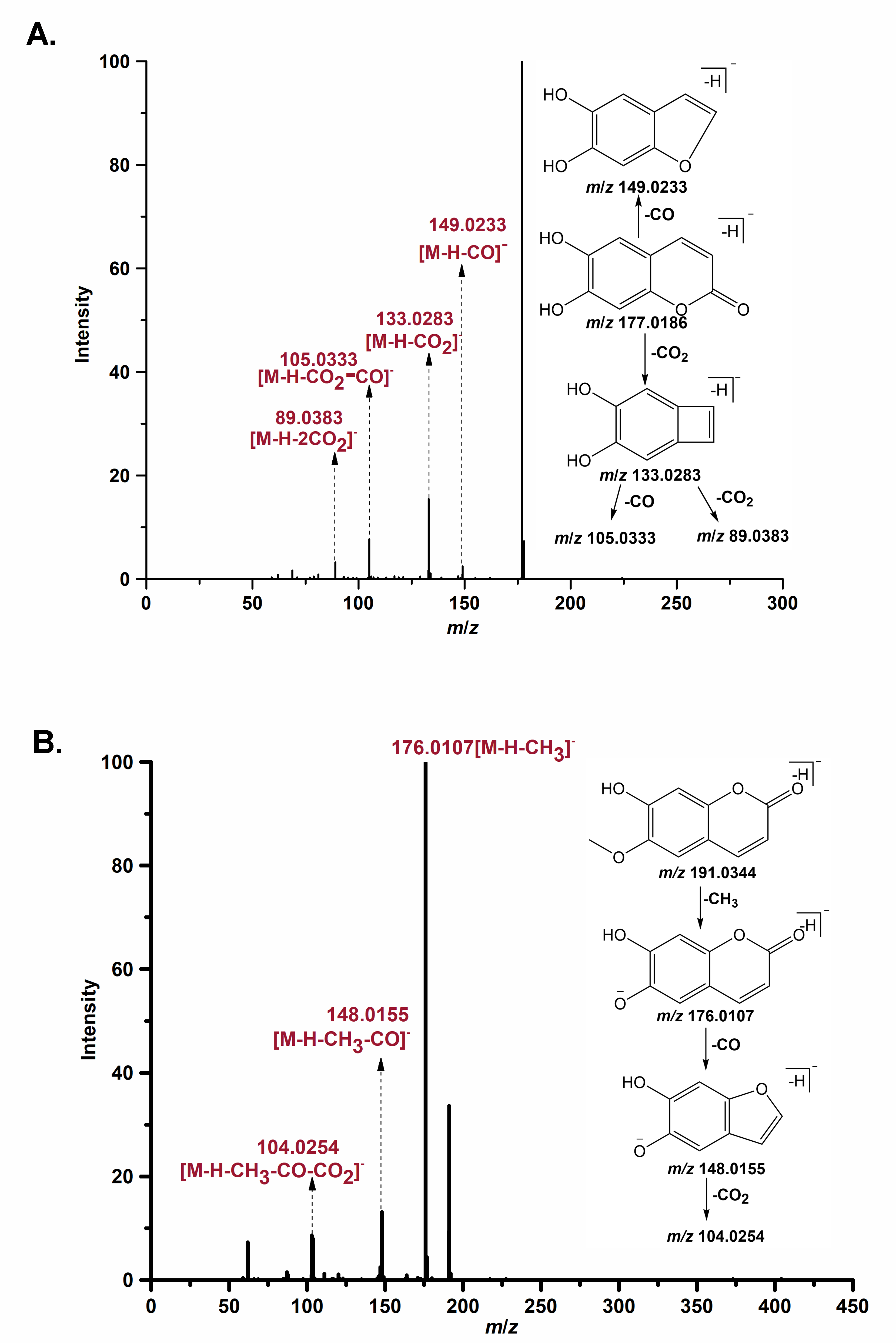


**Supplementary Figure S5.** MS/MS spectra and proposed fragmentation pathways of esculetin (A) and scopoletin (B) from XBSD.

**
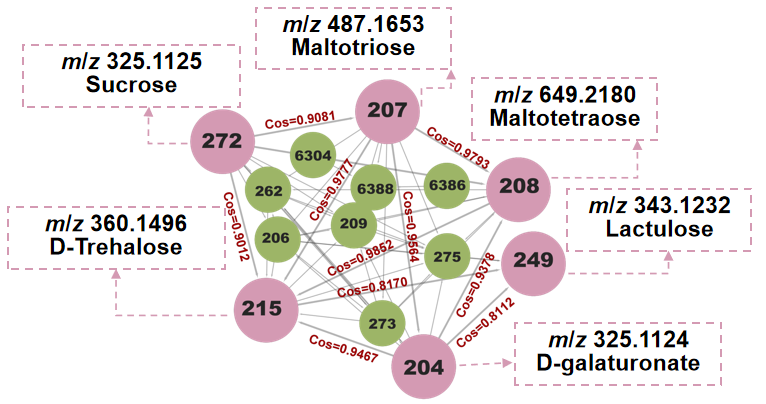
**

**Supplementary Figure S6.** Feature-based molecular networking of saccharides compounds from XBSD.


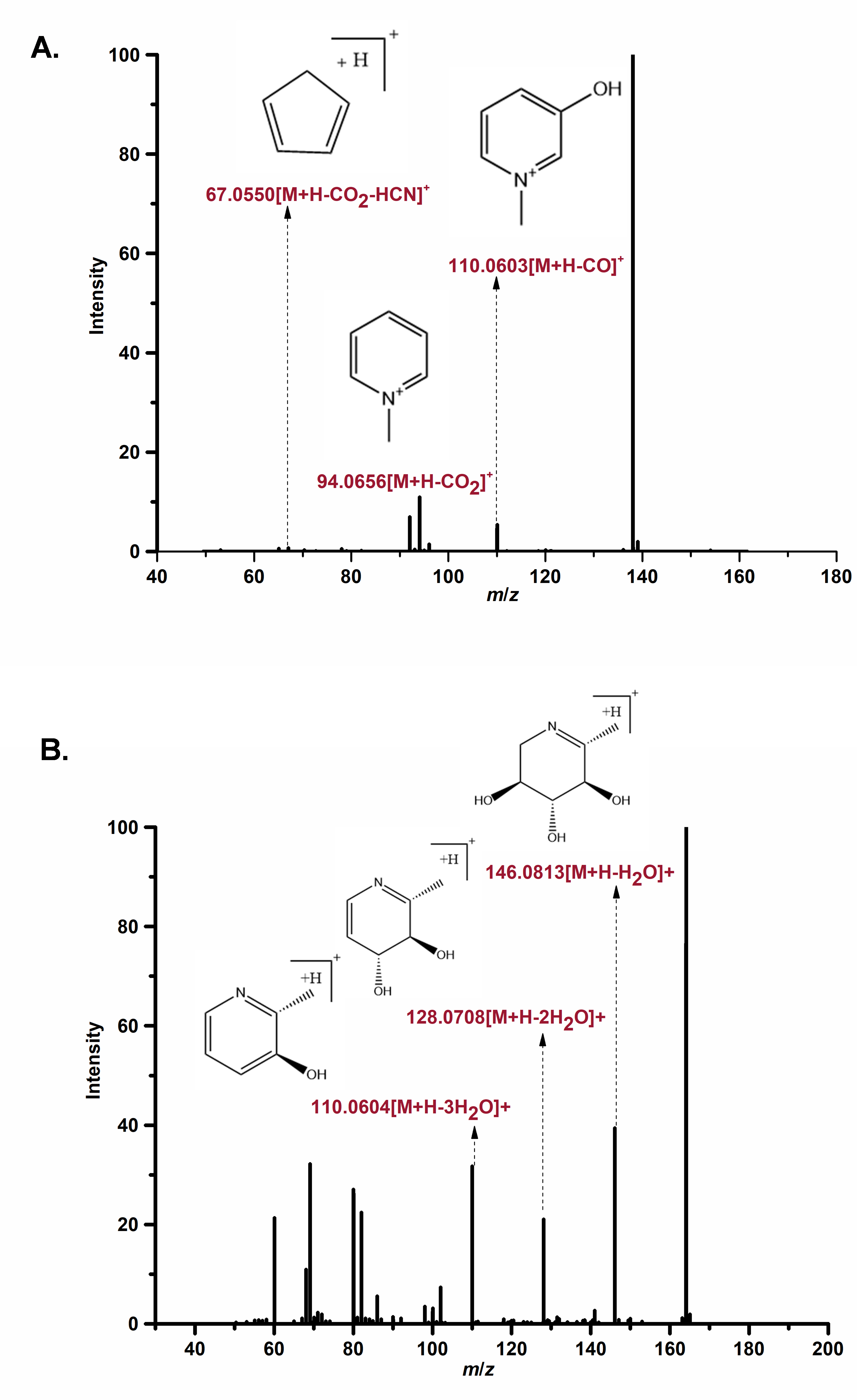


**Supplementary Figure S7.** MS/MS spectra and proposed fragmentation pathways of trigonelline (A) and 1-deoxynojirimycin (B) from XBSD.
